# Kinetic and Products Study of the Atmospheric Degradation of *trans*-2-Hexenal with Cl Atoms

**DOI:** 10.1021/acs.jpca.2c05060

**Published:** 2022-09-27

**Authors:** Asma Grira, María Antiñolo, André Canosa, Alexandre Tomas, Gisèle El Dib, Elena Jiménez

**Affiliations:** † CNRS, IPR (Institut de Physique de Rennes)−UMR 6251, Université de Rennes, F-35000 Rennes, France; ‡ IMT Nord Europe, Institut Mines−Télécom, Univ. Lille, Center for Energy and Environment, F-59000 Lille, France; § Departamento de Química Física, Facultad de Ciencias y Tecnologías Químicas, Universidad de Castilla−La Mancha, Avda. Camilo José Cela 1B, 13071 Ciudad Real, Spain; ∥ Instituto de Investigación en Combustión y Contaminación Atmosférica (ICCA), Universidad de Castilla−La Mancha, Camino de Moledores s/n, 13071 Ciudad Real, Spain

## Abstract

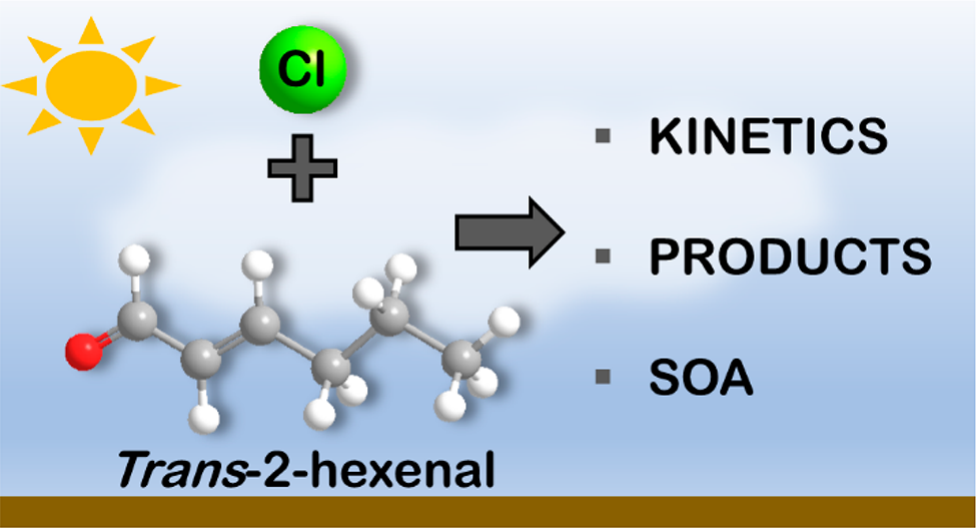

The gas-phase reaction between *trans*-2-hexenal (T2H) and chlorine atoms (Cl) was studied using three complementary experimental setups at atmospheric pressure and room temperature. In this work, we studied the rate constant for the titled oxidation reaction as well as the formation of the gas-phase products and secondary organic aerosols (SOAs). The rate constant of the T2H + Cl reaction was determined using the relative method in a simulation chamber using proton-transfer reaction time-of-flight mass spectrometry (PTR-ToF-MS) to monitor the loss of T2H and the reference compound. An average reaction rate constant of (3.17 ± 0.72) × 10^–10^ cm^3^ molecule^–1^ s^–1^ was obtained. From this, the atmospheric lifetime of T2H due to Cl reaction was estimated to be 9 h for coastal regions. HCl, CO, and butanal were identified as primary products using Fourier transform infrared spectroscopy (FTIR). The molar yield of butanal was (6.4 ± 0.3)%. Formic acid was identified as a secondary product by FTIR. In addition, butanal, 2-chlorohexenal, and 2-hexenoic acid were identified as products by gas chromatography coupled to mass spectrometry but not quantified. A reaction mechanism is proposed based on the observed products. SOA formation was observed by using a fast mobility particle sizer spectrometer. The measured SOA yields reached maximum values of about 38% at high particle mass concentrations. This work exhibits for the first time that T2H can be a source of SOA in coastal atmospheres, where Cl concentrations can be high at dawn, or in industrial areas, such as ceramic industries, where Cl precursors may be present.

## Introduction

Unsaturated C_6_ aldehydes are widely emitted by fresh leaves,^1^ vegetables,^2,3^ and fruits.^4−6^ These compounds could also be distributed through insect secretions to distance or attract other species from their surroundings.^7,8^ Recent studies show that unsaturated C_6_ aldehydes have been detected in the cooking fumes of the deep-frying process (150–200 °C) using cooking oils like coconut, safflower, canola, and extra virgin olive oils.^9−12^*Trans*-2-Hexenal (T2H) is one of these biogenic unsaturated C_6_ aldehydes, which was detected from several sources.^13−17^ T2H could be produced from damaged or wounded leaves as a product of the enzymatic activity of hydroperoxide lyase, a component of the lipoxygenase pathway.^7^ T2H emissions from wounded leaves were demonstrated for tea leaves^18^ and tomatoes.^19^ Damaged plants may react by releasing T2H to induce its self-defense and/or to repair the damages.

Once T2H is released into the atmosphere, it is subjected to oxidation reactions, which proceed through hydrogen atom abstraction mainly from the carbonyl group or addition of the oxidant to the double bond. Hydroxyl (OH) radicals are the main T2H oxidant in the troposphere during daytime^20−22^ and NO_3_ radicals are during night-time,^23^ while the O_3_ reaction has been shown to be of minor importance.^24,25^ Besides, the oxidation of T2H by chlorine (Cl) atoms could be a competitive oxidation channel, especially in the marine boundary layer (MBL) of coastal environments at dawn and in the Arctic troposphere during springtime, where the average [Cl]/[OH] ratio could be 1000 times higher than that at usual conditions.^26^ Therefore, the oxidation process of T2H initiated by Cl may enrich the overall budget of free radicals, tropospheric ozone, and other secondary pollutants in the MBL.^27^ To the best of our knowledge, no experimental reaction product study for the T2H + Cl reaction has been found in the literature that confirmed the formation of secondary gaseous pollutants and/or fine particles, the so-called secondary organic aerosols (SOAs).

While the rate constants of the oxidation reaction of T2H with OH radicals, O_3_, and NO_3_ radicals have been reported in several previous works,^20−23,25,28−35^ the reactivity of T2H with Cl atoms has been scarcely studied. To the best of our knowledge, the only experimental study is from Rodríguez et al.^36^ who studied the kinetics at room temperature and atmospheric pressure (air or N_2_) using a relative method and found a rate constant of (1.92 ± 0.22) × 10^–10^ cm^3^ molecule^–1^ s^–1^. This rate constant is surprisingly lower than the one for *trans*-2-pentenal (T2P) determined in our previous work ((2.56 ± 0.83) × 10^–10^ cm^3^ molecule^–1^ s^–1^, ref (25)), despite it containing less potential reaction sites than T2H. Moreover, Rodríguez et al.^36^ is also lower than the one estimated by the SAR (structure–activity relationship) method (3.47 × 10^–10^ cm^3^ molecule^–1^ s^–1^) developed by Teruel et al.^37^ Thus, in the present paper, the kinetics of the Cl-initiated reaction of T2H at atmospheric pressure and room temperature is revisited.

Regarding the reaction mechanism, a theoretical study by Shashikala and Janardanan^38^ recently reported the most likely reaction channels for the first-step reaction in the degradation mechanisms of T2H by tropospheric oxidants (OH, NO_3_, Cl, and O_3_). These authors found that the reaction of T2H with Cl atoms presented the lowest energy barrier among all oxidants. Also, Shashikala and Janardanan noticed that the H-abstraction reaction from the aldehydic group in T2H was favored against the α- or β-addition to the double bond for the T2H reactions with OH and NO_3_.

In the present work, the gas-phase reaction products of the T2H + Cl reaction are identified and quantified for the first time using FTIR spectroscopy and gas chromatography coupled to mass spectrometry (GC-MS), allowing a reaction mechanism to be proposed. Submicron particles can also be formed in the T2H + Cl gas-phase reaction. Knowing that particle size distributions of these particles are of great interest in the study of ambient aerosols, in the present work, the formation of SOAs with electrical mobility diameter between 5.6 and 560 nm has been monitored using a fast mobility particle sizer (FMPS), reporting SOA mass yields for the first time. The atmospheric implications of the studied reaction are discussed in terms of the atmospheric lifetime of T2H and its contribution, based on the detected products, to local air pollution.

## Experimental Systems, Techniques, and Protocols

The work on the T2H + Cl reaction has been partially carried out at IMT Nord Europe in Douai (France) and UCLM in Ciudad Real (Spain). On the one hand, the kinetic measurements were conducted at IMT Nord Europe in an atmospheric simulation chamber (hereafter D-ASC) at 296 ± 2 K and 730 ± 20 Torr of purified air. The experimental system and procedure are briefly described in “Kinetic Study” and in detail in Turpin et al.^39^ On the other hand, the product study was performed at UCLM in Ciudad Real at 296 ± 2 K and 760 ± 20 Torr of synthetic air using a borosilicate White-type cell of 16 L or an atmospheric simulation chamber (hereafter CR-ASC). This setup and the methodology used are succinctly described in “Product Study”, since it has been detailed in Ballesteros et al.^40^ In separate experiments, the 16-L White cell was connected, in a closed circuit, to the CR-ASC and an FMPS spectrometer (TSI 3091) for carrying out the SOA formation study. This apparatus, procedure, and methodology were previously described in detail elsewhere^25,41^ but are concisely explained below in “Monitoring Ultrafine Particles and Determination of the SOA Yield”.

### Kinetic Study

#### Methodology and Experimental Conditions

The Cl kinetics were performed using the relative method in the D-ASC chamber consisting of a 300 L Teflon bag coupled to a proton-transfer time-of-flight mass spectrometer, PTR-ToF-MS (Kore 2e), for monitoring the signals of T2H and isoprene as a reference compound as a function of time. The two following reactions occur simultaneously in the chamber:
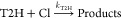
R1
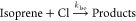
R2where *k*_T2H_ and *k*_Iso_ are the rate constants for T2H + Cl and isoprene + Cl reactions, respectively.

In the PTR-ToF-MS, T2H (C_6_H_10_O) and isoprene (C_5_H_8_) react with a hydronium ion (H_3_O^+^), leading to the molecular ions C_6_H_10_O·H^+^ (*m*/*z* = 99, for T2H) and C_5_H_8_·H^+^ (*m*/*z* = 69, for isoprene) and several protonated fragments: C_4_H_8_·H^+^ (*m*/*z* = 57) and C_6_H_8_·H^+^ (*m*/*z* = 81) for T2H and C_3_H_4_·H^+^ (*m*/*z* = 41) for isoprene. The fragment C_3_H_4_·H^+^ was not used, since its behavior was very different from the C_5_H_8_·H^+^ fragment.

Figure S1 displays an example of the T2H and isoprene fragment evolution during a generic experiment. The experimental procedure started by injecting T2H in the reactor and following its concentration by the PTR-ToF-MS for 40 min in the dark (Figure S1, *t* = 55–95 min). Then, isoprene was added to the reactor and allowed to homogenize for 30 min (Figure S1, *t* = 95–125 min). During that time, two UV actinic lamps (Philips TLK-40 W/05 and Sylvania Blacklight, λ_max_ = 365 nm) were turned on to quantify the losses of T2H and isoprene by photolysis. Then, UV lights were turned off, and molecular chlorine (Cl_2_) was added to the reaction mixture and left for 25 min in the chamber (Figure S1, *t* = 125–150 min). T2H and isoprene losses were evaluated during the described steps to check for possible adsorption on the D-ASC walls, UV photolysis, and/or a dark reaction with Cl_2_. Negligible losses were observed for both T2H and isoprene with a ratio of the standard deviation to the mean value of the concentration within ∼45 min of measurements of less than 1.1%. Then, the lamps were switched on for 30 to 40 min in the presence of the T2H + isoprene + Cl_2_ mixture (Figure S1, *t* = 150–190 min). At the end of each experiment, the reactor was cleaned by filling it with air and pumping it up several times.

The initial concentrations of T2H and isoprene in the D-ASC chamber were determined by a Fourier transform infrared, FTIR, spectrometer (Nicolet 6700 Thermo Fisher) using the IR spectral features in the 1182–1116 cm^–1^ range for T2H and in the 943–844 cm^–1^ range for isoprene. The quantification was done by using a reference spectrum of each reagent. The initial concentration of Cl_2_ was calculated based on its injected volume into the chamber as explained in eq S1. The initial concentrations of T2H, isoprene, and Cl_2_ were in the ranges (5.9–11.6) × 10^13^ molecules cm^–3^, (6.4–13.3) × 10^13^ molecules cm^–3^, and (14.5–58.3) × 10^13^ molecules cm^–3^, respectively.

#### Determination of the Rate Constant *k*_T2H_

In the presence of Cl atoms, the relative disappearance rates of T2H and isoprene were measured. The time evolution of the concentrations of T2H and the reference compound is described by the following expression, since there are no secondary loss processes:
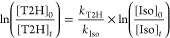
1[T2H]_0_, [Iso]_0_ and [T2H]_*t*_, [Iso]_*t*_ are the concentrations of T2H and isoprene at reaction times *t* = 0 and *t*, respectively. The ratio of rate constants *k*_T2H_/*k*_Iso_ was obtained by plotting ln([T2H]_0_/[T2H]_*t*_) versus ln([Iso]_0_/[Iso]_*t*_). *k*_T2H_ is then derived from the slope of such plots considering *k*_Iso_ as (4.6 ± 0.9) × 10^–10^ cm^3^ molecule^–1^ s^–1^, which is the weighted average of three values found in the literature^42−44^ (see Table S2). *k*_Iso_ and the overall error (Δ*k*_Iso_) were calculated using eqs S2–S4. In this work, we considered Δ*k*_Iso_ as twice the standard deviation of the weighted average *k*_Iso_, 2σ_<kIso>_.

The overall error in *k*_T2H_, Δ*k*_T2H_, was calculated according to eq S5 and it comprises statistical errors, Δ*k*_T2H_(stat), and systematic errors, Δ*k*_T2H_(syst). Δ*k*_T2H_(stat) was calculated according to eq S6, and Δ*k*_T2H_(syst) was estimated to be 10%.

### Product Study

#### Procedure and Experimental Conditions

The experimental procedure started with the injection of T2H in the reactor (16-L or 264-L CR-ASC). In the 16-L reactor, the concentration of T2H was determined using FTIR spectroscopy (Nicolet Nexus 870, Thermo) based on a reference IR spectrum. IR spectra were recorded every 2 min with a resolution of 2 cm^–1^ in the IR spectral range 4000–650 cm^–1^ by accumulating 32 interferograms. In separate experiments in CR-ASC, the solid phase micro extraction (SPME)/GC-MS technique was used. In these experiments, a 50/30 μm divinylbenzene/Carboxen polydimethylsiloxane fiber (Supelco) was inserted every 15 min in the CR-ASC to absorb analytes for 5 min and injected in the GC-MS (Trace GC Ultra and DSQ II, Thermo Electron) equipped with a BPX35 column (30 m × 0.25 mm ID × 0.25 μm, SGE Analytical Science) working at a temperature ramp that ranged between 40 and 250 °C.

Actinic lamps (Philips Actinic BL TL 40W/10 1SL/25 (16-L reactor) and Philips TLK – 40 W/05 (CR-ASC), λ = 340–400 nm) were used to irradiate the gas sample with UV radiation, which generate Cl atoms from Cl_2_ photolysis. In both reactors, after stabilization for 30 min, UV lights were turned on for 30 min to test the stability of T2H under photolysis conditions. Cl_2_ was then added to the reactor, and the reaction mixture (synthetic air + T2H + Cl_2_) was left to stabilize for 30 min in the dark. The loss rate constant of T2H due to heterogeneous and dark reactions and UV photolysis was on the same order of magnitude (<4 × 10^–5^ s^–1^). Finally, when UV lights were turned on, the T2H + Cl reaction began, and the gaseous products formed were monitored for 60 min. The concentrations of T2H and Cl_2_ used for the product studies are summarized in Table 1.

**Table 1:** Experimental Conditions and Techniques Used during the Product Study

reactor (number of runs)	analytical technique	number of lamps (model)	compound	initial concentration range (10^14^ molecules cm^–3^)
16-L (7)	FTIR	3 (Philips Actinic BL TL 40 W/10 1SL/25)	T2H	4.7–22
			Cl_2_	3.8–13
CR-ASC (2)	SPME/GC-MS	8 (Philips TLK–40 W/05)	T2H	6.2–6.3
			Cl_2_	4.1

#### Determination of the Product Yield (*Y*_Prod_)

Two types of products can be distinguished: primary products directly formed through the T2H + Cl oxidation reaction and secondary products generated by primary product degradation. The product yield (*Y*_Prod_) of a primary product is defined as the ratio of its concentration formed at a certain reaction time in the T2H + Cl reaction, [Prod], to that of the consumed reactant, Δ[T2H], over the same time scale according to eq 2.
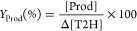
2Therefore, the [Prod] vs Δ[T2H] plot must be linear to guaranty that the product has a primary origin, and the slope represents *Y*_Prod_.

#### Monitoring Ultrafine Particles and Determination of the SOA Yield

To monitor the particle formation in the T2H + Cl reaction, the CR-ASC, the 16-L reactor, and the FMPS spectrometer were coupled. The FMPS spectrometer analyzes the particle number size distribution (electrical mobility diameters between 5.6 and 560 nm with a time resolution of 1 s) averaged over 1 min. The initial concentrations of T2H and Cl_2_ were in the ranges (0.8–2.7)×10^14^ molecules cm^–3^ and (1.3–7.2)×10^14^ molecules cm^–3^, respectively.

The procedure was as follows: the gas mixture was recirculated in a closed circuit allowing the gases to mix in the system. The system allows to simultaneously monitor the loss of T2H by in situ IR in the 16-L chamber and the FMPS sampling. The gas mixture flows from the CR-ASC to the FMPS through a 1 μm cut cyclone at a 10 L min^–1^ flow rate (which is the sampling flow rate of the instrument), and the filtered exhaust from the FMPS was then directed to the 16-L reactor where IR measurements were performed every 2 min by means of the FTIR spectrometer described in section “Kinetic Study”. Then, the gas mixture comes back to the CR-ASC closing the circuit. Indeed, due to the high sampling flow rate of the FMPS, it cannot be directly connected to the 16-L chamber. This system was previously used in the study of the T2P + Cl reaction by Grira et al.^25^

The total time scale of the experiment was typically 80 min. In the first 10 min, the reaction mixture (synthetic air + T2H + Cl_2_) was left in the dark, and the potential T2H wall losses and reaction with Cl_2_ were estimated. While no reaction between T2H and Cl_2_ was observed, a T2H wall loss constant *k*_L,T2H_ of <4 × 10^–5^ s^–1^ was determined in the dark. *k*_L,T2H_ was determined from the decay of [T2H] as exemplified in Figure S2a. Once the lamps surrounding the CR-ASC were turned on, the Cl reaction started, and T2H consumption and SOA formation were monitored for typically 50 min. SOA wall losses onto the reactor walls and/or through the filters of the FMPS, *k*_L,SOA_, were evaluated leaving the reaction mixture for typically 20 min in the dark, after the end of the reaction. This parameter was obtained from the decay of the mass concentrations of particles (*M*_SOA_) shown in the example depicted in Figure S2b. *M*_SOA_ was calculated using the measured particle number size distributions assuming spherical geometry and a particle density of 1.4 g cm^–3^ (recommended by Hallquist et al.^45^). The loss rate constant *k*_L,SOA_ was measured to be in the range (4.35–6.18) × 10^–4^ s^–1^. From the slope of *M*_SOA_ versus T2H consumption (Δ[T2H]) plots, as shown by eq 3, the SOA yield (*Y*_SOA_) can be determined. Both *M*_SOA_ and Δ[T2H] were corrected as described in previous works, Antiñolo et al.^46^ and Grira et al.,^25^ taking into account *k*_L,T2H_, and *k*_L,SOA_.
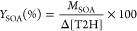
3

### Chemicals

The chemicals and gases employed in this work at IMT were the following ones (in bracket the purity and supplier are stated): T2H (98%, Sigma–Aldrich), isoprene (99%, Sigma–Aldrich), Cl_2_ (10% in N_2_, Air Products). At IMT, we used an extra pure zero air, which was produced with a pure air generator (AZ-2020, Claind) with relative humidity (RH) < 2 ppm, and CO and CO_2_ < 80 ppb. At UCLM, T2H (98%, Sigma–Aldrich), Cl_2_ (99.8%, Sigma–Aldrich), and synthetic air (99.999%, Air Liquide) was used.

## Results and Discussion

### Rate Constant for the T2H + Cl Reaction

**Figure 1:**
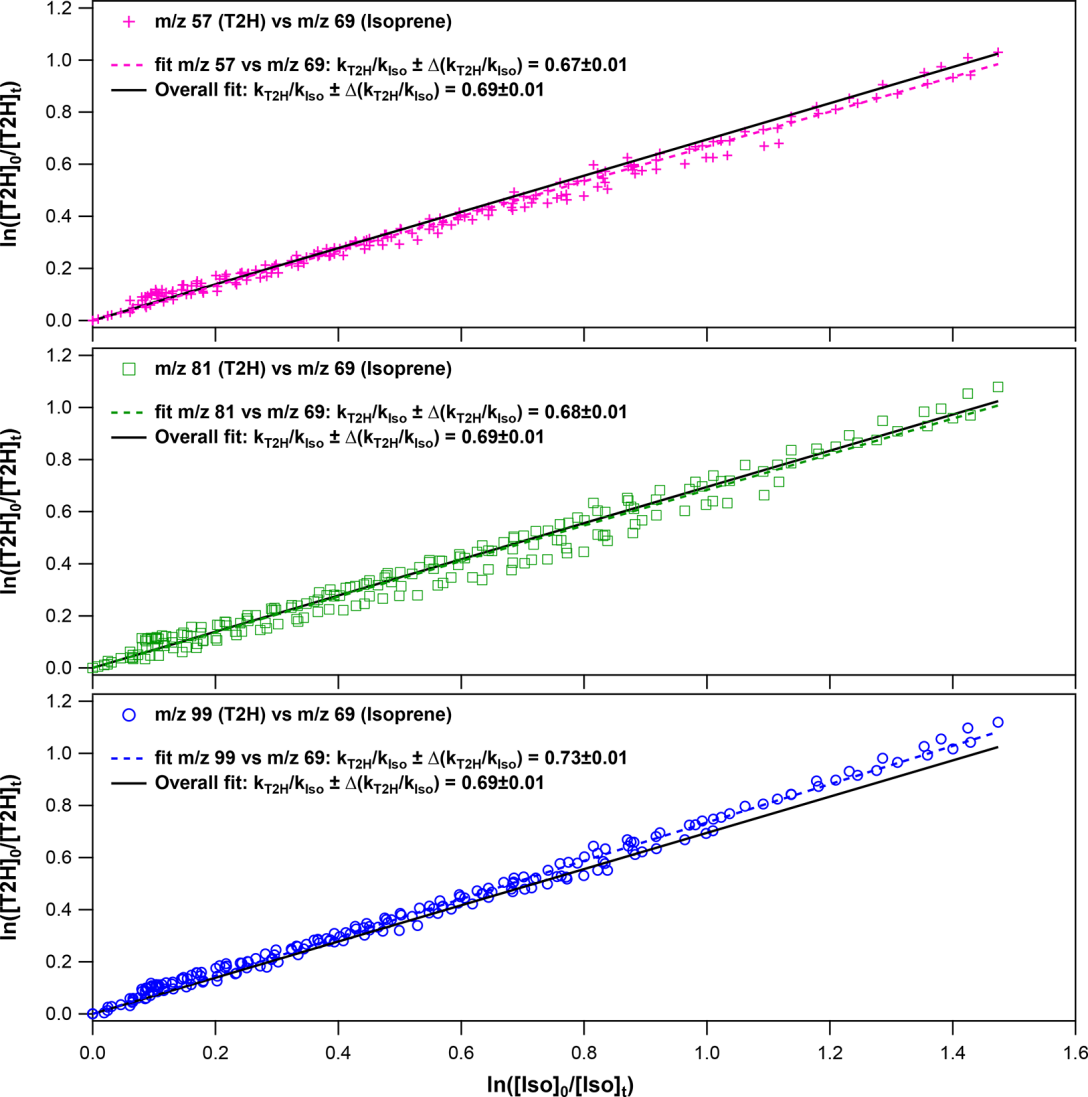
Plots of the decay of [T2H] vs that of [isoprene] in the presence of Cl for each observed peak of T2H. The continuous black line in each panel shows the linear regression of the combined data from the three series.

Figure 1 displays the obtained loss of T2H versus that of the reference compound in the presence of Cl atoms. The plot of ln([T2H]_0_/[T2H]_*t*_) versus ln([Iso]_0_/[Iso]_*t*_) displays a good linearity, and the ratios *k*_T2H_/k_Iso_ are relatively consistent for the three masses of T2H used in the analysis (*m*/*z* = 99, *m*/*z* = 81, and *m*/*z* = 57). The reported slope *k*_T2H_/*k*_Iso_ (0.69 ± 0.01) arises from a linear regression of the combined data from the three series. *k*_T2H_ was determined from *k*_T2H_/*k*_Iso_ considering the *k*_Iso_ given in section “Kinetic Study”:
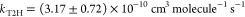
The overall error in the rate constant is 23% (see Supporting Information for a detailed explanation of the error analysis).

#### Comparison with Literature Data

Table 2 gathers the *k*_T2H_ obtained in this work on the T2H + Cl reaction and that reported by Rodríguez et al.^36^ As it can be seen, this latter value of *k*_T2H_ is 39% lower than the present measurement. In Rodríguez et al.’s work, the monitoring of concentration of T2H and the reference compounds (ethane and propene) was carried out by GC coupled to a flame ionization detector (GC-FID). The source of the observed discrepancy is not due to Cl rate constants used by these authors for the reference compounds (*k*_Ethane_ and *k*_Propene_). In 2009, IUPAC updated the recommended values of *k*_Ethane_ and *k*_Propene_ for the rate constants of the Cl-initiated reactions of ethane and propene. This re-evaluation of *k*_T2H_ slightly changes the value by Rodríguez et al. by about 7%: (2.06 ± 0.46) × 10^–10^ cm^3^ molecule^–1^ s^–1^.

**Table 2:** Summary of the Rate Constants for the Reactions of a Series of C_3_–C_7_ Alkenals with Cl Atoms at Room Temperature and Atmospheric Pressure

unsaturated aldehyde (linear formula)	*k* (10^–10^ cm^3^ molecule^–1^ s^–1^)	technique^a^	reference
2-propenal or acrolein (CH_2_=CH–CHO)	2.2 ± 0.3	GC-FID	Thévenet et al.^47^
	2.2 ± 0.3	FTIR	Canosa-Mas et al.^48^
	1.8 ± 0.3	FTIR	Ullerstam et al.^49^
	2.5 ± 0.7	GC-FID	Wang et al.^50^
2-methyl-2-propenal or methacrolein (CH_2_=C(CH_3_)–CHO)	3.2 ± 0.5	FTIR	Canosa-Mas et al.^48^
	2.9 ± 0.8	GC-FID	Wang et al.^50^
*trans*-2-butenal or crotonaldehyde (*trans*-CH_3_–CH=CH–CHO)	2.6 ± 0.3	GC-FID	Thévenet et al.^47^
	2.2 ± 0.4	FTIR	Ullerstam et al.^49^
	3.2 ± 0.9	GC-FID	Wang et al.^50^
*trans*-2-methyl-2-butenal (*trans*-CH_3_–CH=C(CH_3_)–CHO)	2.45 ± 0.32	FTIR	Antiñolo et al.^41^
*trans*-2-pentenal (*trans*-CH_3_–CH_2_–CH=CH–CHO)	1.31 ± 0.19	GC-FID	Rodríguez et al.^36^
	3.47	SAR estimation	this work
	2.56 ± 0.83	FTIR	Grira et al.^25^
*trans*-2-hexenal (*trans*-CH_3_–CH_2_–CH_2_–CH=CH–CHO)	1.92 ± 0.22	GC-FID	Rodríguez et al.^36^
	2.06 ± 0.46	re-evaluation in the present work	Rodríguez et al.^36^
	3.17 ± 0.72	PTR-MS	this work
*trans*-2-heptenal (*trans*-CH_3_–CH_2_–CH_2_–CH_2_–CH=CH–CHO)	2.40 ± 0.29	GC-FID	Rodríguez et al.^36^

a GC-FID: Gas chromatography-flame ionization detection; FTIR: Fourier transform infrared; PTR-MS: proton-transfer mass spectrometry.

In Figure S3, a comparison between the rate constants determined in different studies for the Cl reaction of a series of C_3_–C_7_ alkenals is presented. It is noted that data from Rodríguez et al.^36^ are systematically lower than other studies, indicating that they may probably have unaccounted systematic errors. Therefore, the source of the observed discrepancy is still unclear.

#### Structure–Activity Relationship (SAR)

Using the method proposed by Teruel et al.,^37^ which is based on a basic rate constant for the *trans*-RHC=CHR structure and some “group factors”, the rate constant for the T2H + Cl can be estimated. Teruel et al.^37^ provides the value of the group factor for −C(O)H but not for the CH_3_CH_2_CH_2_– group. Instead, the value for the CH_3_CH_2_– group is given. Since it is expected that the contribution of the CH_3_CH_2_CH_2_– group to the estimated *k*_T2H_ is higher than that of the CH_3_CH_2_– group, it is likely that the corresponding group factor is at least equal to the CH_3_CH_2_– one. Hence, a lower limit of the *k*_T2H_ estimated with the SAR method can be provided: *k*_T2H(SAR)_ = 3.47 × 10^–10^ cm^3^ molecule^–1^ s^–1^. This number is very close to the experimental value reported here and 83% higher than that reported by Rodríguez et al.^36^

When comparing the Cl reactivity of linear unsaturated aldehydes, we observe that the rate constant for Cl reactions with C_3_–C_7_ alkenals seems to depend barely on the aldehyde chain length (Table 2 and Figure S3). As shown in Figure S3 and excluding the result for *trans*-2-heptenal reported by Rodríguez et al.,^36^ the Cl rate coefficient increases by about 50% from 2-propenal (*C*_3_) to *trans*-2-hexenal (*C*_6_), even though there is some scattering in the literature values.

Comparing the Cl reactivity toward *trans*-2-butenal and *trans*-2-pentenal with that of branched unsaturated aldehydes with the same number of carbon atoms, such as 2-methyl-2-propenal and *trans*-2-methyl-2-butenal, we observed that the presence of the methyl group has no apparent effect on the reactivity. No data are available up to date to compare the reactivity of *trans*-2-hexenal with a branched unsaturated aldehyde with the same number of C atoms.

It would have been interesting to compare the reactivity of T2H toward Cl atoms with that of the corresponding alkene, since it has been postulated that alkenes and their corresponding unsaturated aldehydes react via similar Cl addition to the double bond.^51^ Unfortunately, no data is available in the literature for the reaction of Cl with *trans*-2-hexene. Further experimental studies on alkenes + Cl reactions would be desirable.

### Gaseous Reaction Products

#### Temporal Evolution of Species in the Reaction Mixture

##### Detection by FTIR Spectroscopy

**Figure 2:**
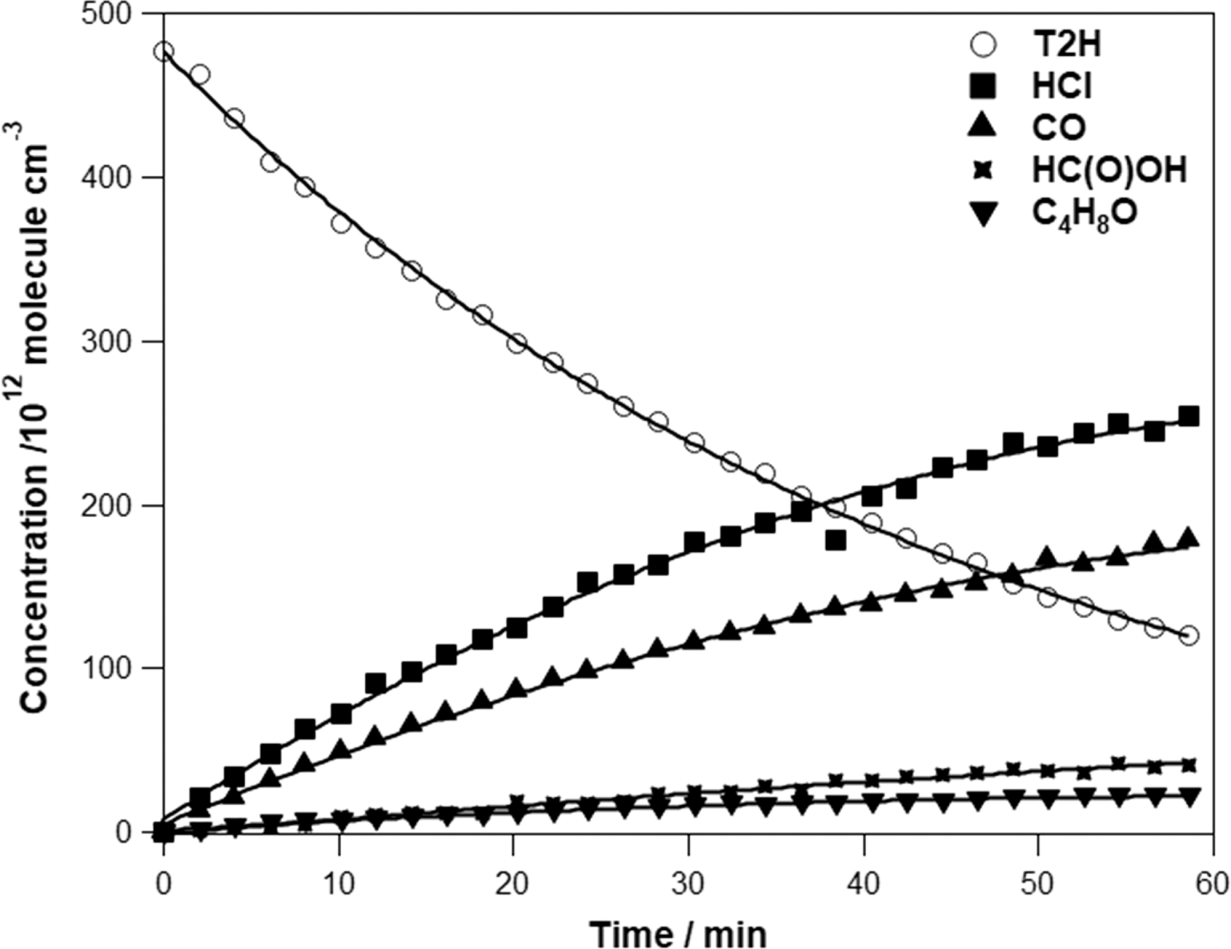
Concentration–time profile for T2H and the major products using [T2H]_0_ = 4.7 × 10^14^ molecules cm^–3^ and [Cl_2_]_0_ = 3.9 × 10^14^ molecules cm^–3^.

As can be seen in Figure S4, IR features of HCl, CO, HC(O)OH, and butanal are present in the final spectrum. The IR spectral features used for this analysis were 1610–1750 cm^–1^ for T2H, 2600–3100 cm^–1^ for HCl, 2039–2239 cm^–1^ for CO, 2870–2950 cm^–1^ for HC(O)OH, and 2750–2660 cm^–1^ for butanal. The identification and the quantification of these products were made using reference IR spectra. After subtraction of the IR features of the products, the residual spectrum showed some peaks that indicate that there were unidentified products. The group of peaks between 3000 and 2700 cm^–1^ corresponds to C–H stretching vibrational modes. The most intense peak centered at 1750 cm^–1^ is typically assigned to C=O bonds, and the weak bands between 1500 and 1000 cm^–1^ can be due to C–C stretching or C–H bending modes. Figure 2 shows typical concentration vs time profiles for the consumption of T2H and the formation of the detected products.

##### Detection by SPME/GC-MS

As shown in Figure S5, the reaction products detected by GC-MS were butanal (retention time RT = 2.88 min), 2-chlorohexanal (RT = 4.84 min), and 2-hexenoic acid (RT = 6.36 min). They were identified by means of their electronic impact mass spectra. However, the signal from the GC-MS was not stable enough to allow for a reliable quantification of these species, and no internal standard was introduced in the gas mixture.

The products detected in this work are in agreement with those observed for the crotonaldehyde + OH reaction^44^ and *trans*-2-pentenal + Cl reaction.^25^ In all these reactions, the corresponding saturated aldehyde with two less C atoms and CO were formed.

#### Molar Yields of Reaction Products

**Figure 3:**
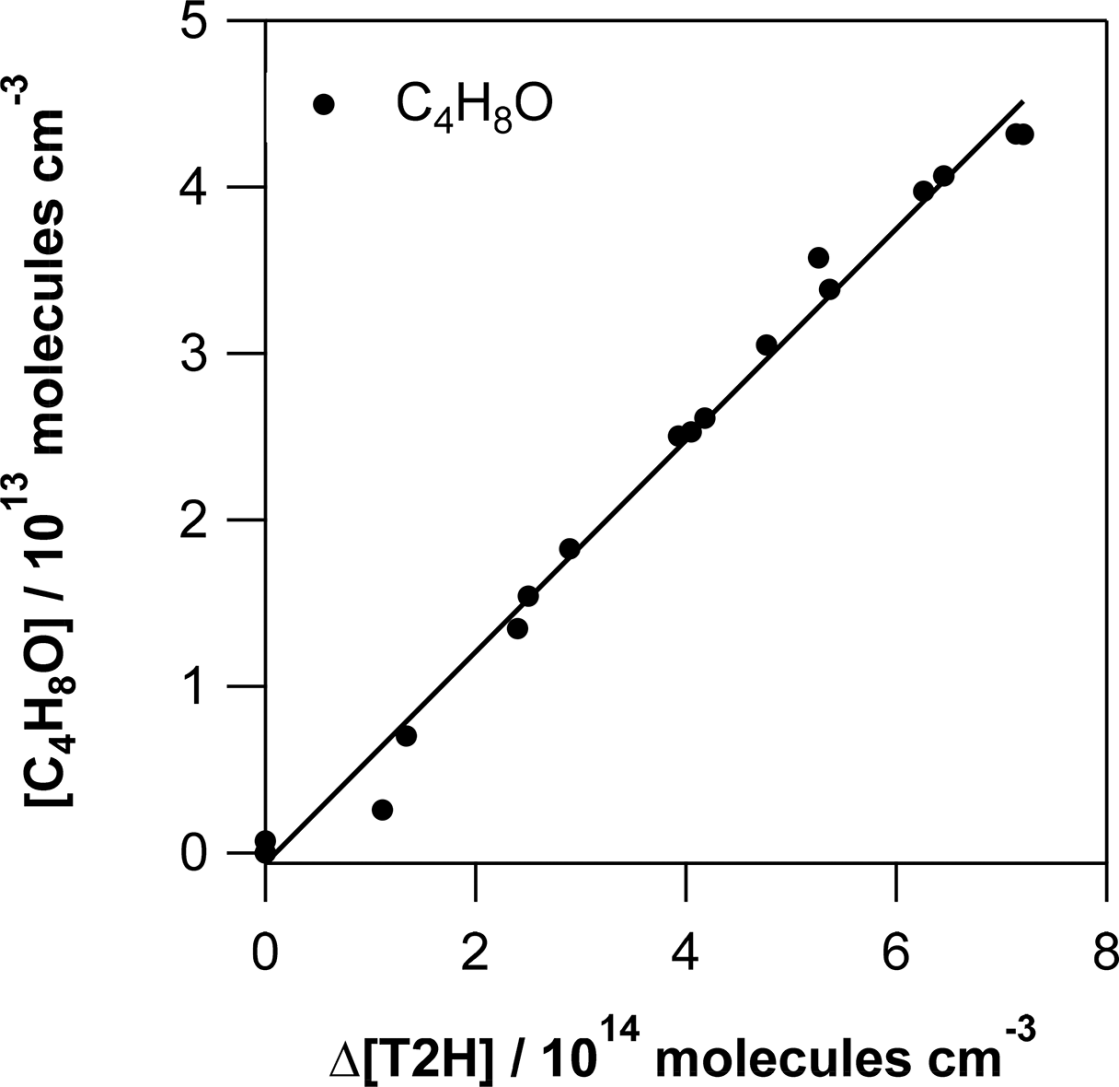
Product yield for butanal.

The product yields were determined based on the quantified concentrations of T2H and products by FTIR spectroscopy as a function of time. Here, only the butanal yield is reported, since the other quantified products (HCl, CO, and HC(O)OH) are known to be very final products with a wide variety of sources, and their molar yields provide little information. The corresponding plot displaying [Butanal] vs Δ[T2H] according to eq 2 is shown in Figure 3. The overall errors on the formation yields (Δ*Y*_Prod_) were calculated as described in eq S7. The molar yield of butanal obtained from the slope is (6.35 ± 0.30)%. The carbon mass balance was found to be more than 20% at the end of the reaction, if calculated considering the formation of butanal, CO, and HC(O)OH.

#### Proposed Reaction Mechanism

**Figure 4:**
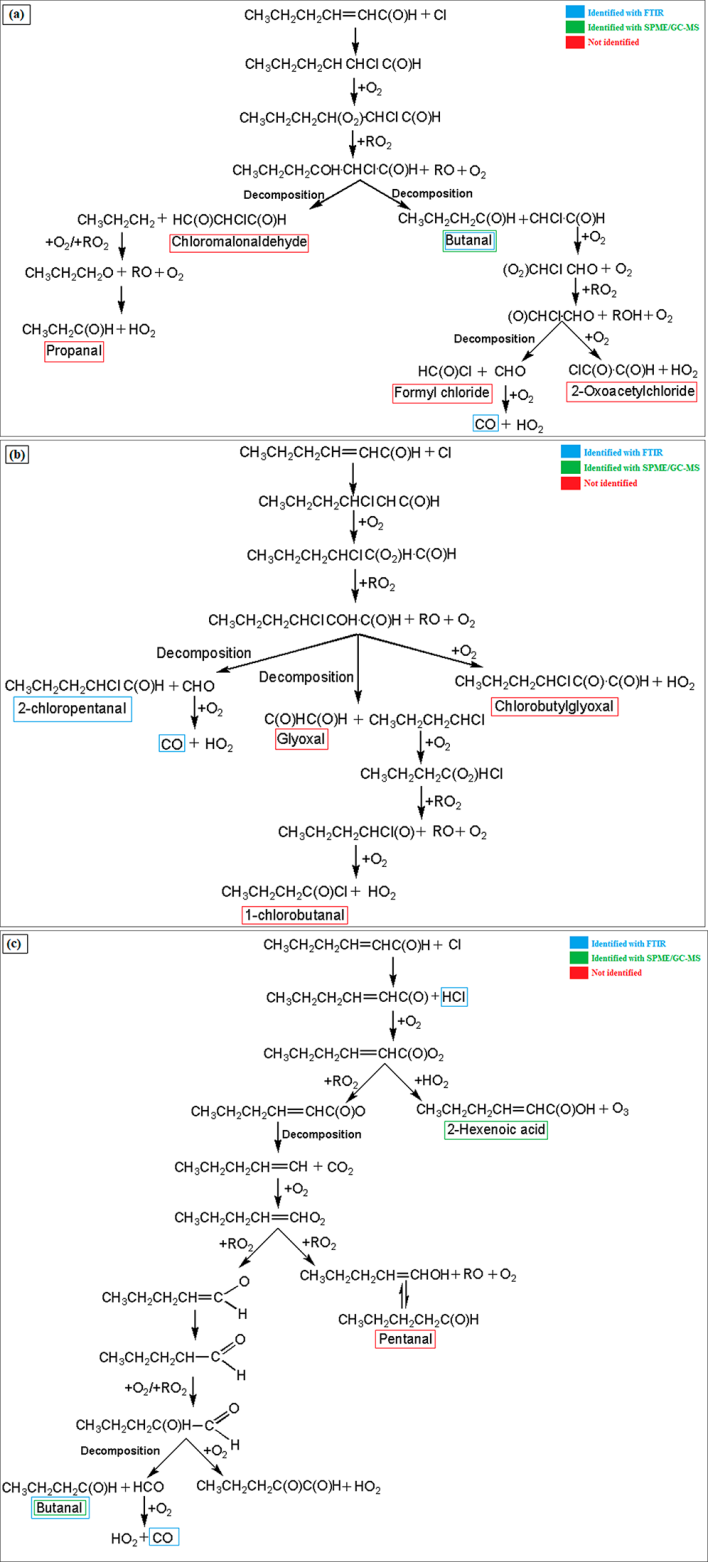
Proposed mechanisms for the reaction of T2H with a Cl atom: α-addition (a), β-addition (b), and H-abstraction (c).

Based on previous experimental studies on the Cl reaction of unsaturated aldehydes^25,52^ and the theoretical work of Shashikala and Janardanan on the T2H degradation,^38^ the reaction is expected to proceed via three pathways: addition of the Cl atom to the C=C double bond, either to C_α_ or C_β_, and abstraction of the hydrogen atom from the −C(O)H group. Shashikala and Janardanan^38^ concluded that allylic H-abstraction is a minor reaction pathway; therefore, in this work, this route has not been considered. In Figure 4, the resulting reaction pathways that may support the observed products are displayed.

As seen in Figure 4a, the α-addition forms the CH_3_CH_2_CH_2_CHCHClC(O)H radical, which can react with O_2_/RO_2_ to lead to the corresponding chloroalkoxy radical (CH_3_CH_2_CH_2_COHCHClC(O)H). The decomposition of the chloroalkoxy radical leads to the formation of butanal (CH_3_CH_2_CH_2_C(O)H) and, after further reactions, 2-oxoacetyl chloride (ClC(O)C(O)H) and formyl chloride (HC(O)Cl). CO is also formed as a coproduct of formyl chloride. The second channel leads to the formation of propanal (CH_3_CH_2_C(O)H) and chloromalonaldehyde (HC(O)CHClC(O)H), which were not detected.

In Figure 4b, the β-addition mechanism proceeds through the formation of the CH_3_CH_2_CH_2_CHClCHC(O)H radical, which can react with O_2_/RO_2_ to lead to the CH_3_CH_2_CH_2_CHClCOHC(O)H radical. This radical may decompose to lead to the formation of 2-chloropentanal (CH_3_CH_2_CH_2_CHClC(O)H) and CO, identified by FTIR. As shown in Figure 4b, the CH_3_CH_2_CH_2_CHClCOHC(O)H radical may also lead to the formation of glyoxal (C(O)HC(O)H), 1-chlorobutanal (CH_3_CH_2_CH_2_C(O)Cl), and chlorobutylglyoxal (CH_3_CH_2_CH_2_CHClC(O)C(O)H), which were not detected in this work, suggesting that either these channels do not occur or these routes are minor with respect to others.

In Figure 4c, the H-abstraction from the formyl group leads to the formation of the CH_3_CH_2_CH_2_CH=CHC(O) radical and HCl, identified by FTIR. In the presence of O_2_, the CH_3_CH_2_CH_2_CH=CHC(O) radical may be transformed to the acylperoxy radical CH_3_CH_2_CH_2_CH=CHC(O)O_2_, which produces 2-hexenoic acid (CH_3_CH_2_CH_2_CH=CHC(O)OH) (identified by GC-MS) and ozone in the presence of HO_2_. The CH_3_CH_2_CH_2_CH=CHC(O)O_2_ radical may also react with RO_2_ and produce the CH_3_CH_2_CH_2_CH=CHC(O)O radical, which decomposes rapidly into CO_2_ and the CH_3_CH_2_CH_2_CH=CH radical. The latter is converted to CH_3_CH_2_CH_2_CH=CHO_2_ in the presence of O_2_ further reacting with RO_2_ to generate pentanal CH_3_CH_2_CH_2_CH_2_C(O)H (not identified), butanal CH_3_CH_2_CH_2_C(O)H (identified by FTIR and GC-MS and quantified by FTIR), and CO (identified by FTIR).

In the three proposed routes, the RO_2_ radicals can undergo autoxidation leading to the formation of peroxides and hydroperoxides that are highly oxygenated organic molecules (HOMs). The RO_2_ autoxidation reaction consists of an intramolecular H-abstraction or a H-shift, that generates an alkyl radical, R, with a hydroperoxyl functional group (−OOH). For example, in the Cl + T2H reaction, the first RO_2_ radical formed in Figure 4a can be autoxidized to
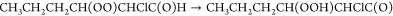
R3The CH_3_CH_2_CH_2_CH(OOH)CHClC(O) radical can further react with O_2_ to form a new, more oxidized, RO_2_:
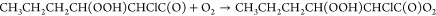
R4The CH_3_CH_2_CH_2_CH(OOH)CHClC(O)O_2_ radical will further react probably leading to the formation of HOMs. HOMs are known to condense easily due to their low vapor pressure and could not be detected in this work but they may contribute to particle formation.

In the proposed mechanism, since CO is formed from the three channels, and butanal is part of the α-addition and the H-abstraction routes, these compounds are not of great help to quantify the relative contributions of addition and abstraction channels. On the other hand, HCl and 2-hexenoic acid were observed only in H-abstraction. However, HCl can also be issued from other abstraction reactions occurring in the reactor, and then, its yield may not reflect the one of the T2H + Cl reaction alone. The experimental observations, nevertheless, indicate that all three channels should be open. Although formic acid has been identified in the present work, it does not appear in the three suggested mechanisms. Therefore, it is likely that it is generated from secondary processes in the reactor.

### Particle Formation

#### Particle Size Distribution and Total Number Concentration

**Figure 5:**
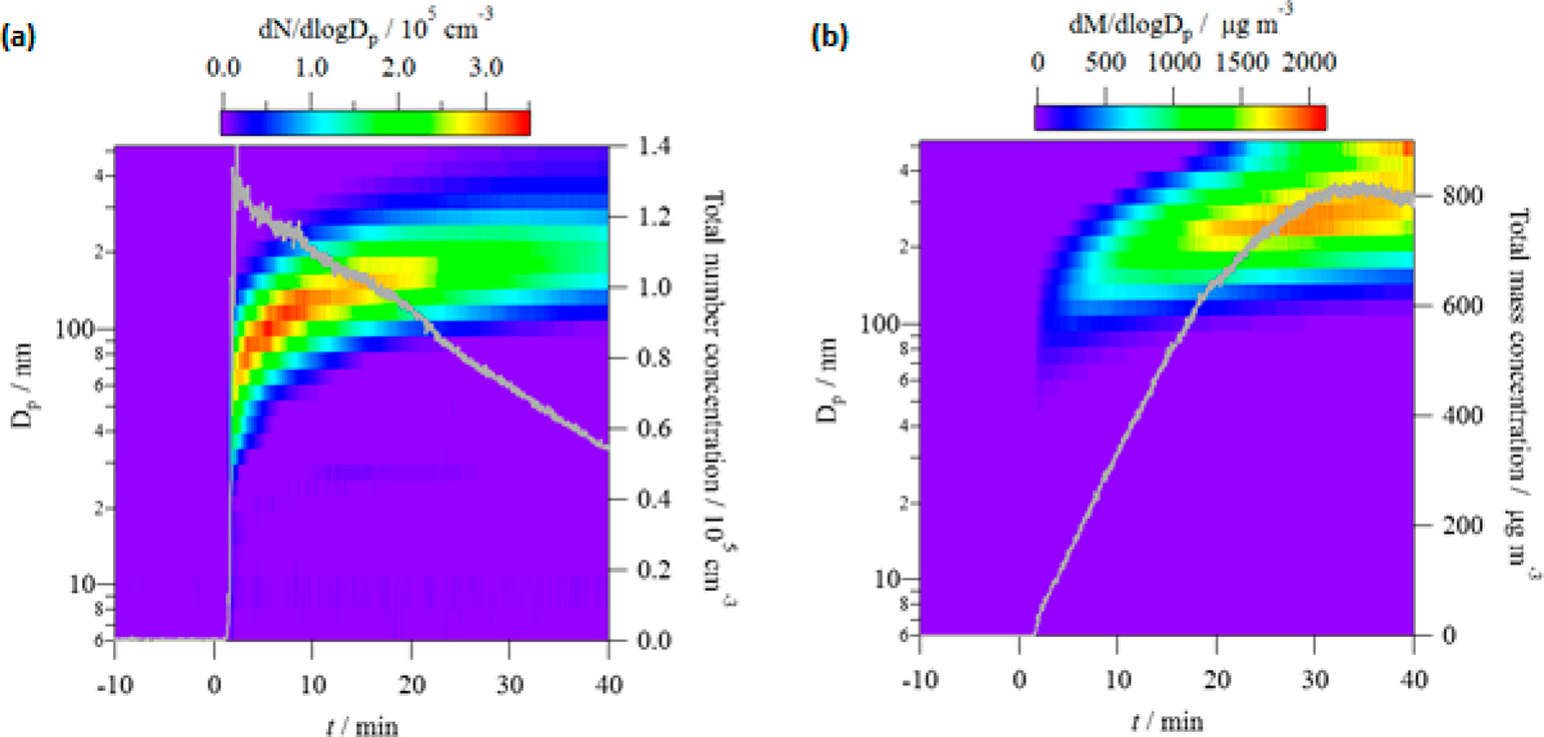
Typical time-dependent size distributions for SOA from T2H + Cl measured by the FMPS in terms of (a) particle number concentrations and (b) particle mass concentrations, considering a mass density of 1.4 g cm^–3^. Initial concentrations were 2.7 × 10^14^ and 6.5 × 10^14^ molecules cm^–3^ for T2H and Cl_2_, respectively. Time zero corresponds to the start of the reaction. The gray line represents the temporal evolution of the total (a) number concentration and (b) mass concentration.

A typical temporal evolution of the particle size distribution, in terms of normalized particle number concentrations (d*N*/dlogDp) is displayed in Figure 5a for given initial concentrations of T2H and Cl_2_. In this example, at *t* = 2 min, the first particles appeared rapidly reaching a total number concentration (*N*_p_) around 1.4 × 10^5^ particles cm^-3^ with the maximum at about 70 nm diameter. From *t* = 2 min to *t* = 10 min, the diameter of the maximum grew from 70 to 143 nm, while *N*_p_ decreased down to 1.0 × 10^5^ particles/cm^3^. Finally, from *t* = 10 min to *t* = 40 min (end of the reaction), *N*_p_ decreased to 5.4 × 10^4^ particles cm^–3^, and the maximum diameter increased up to 221 nm. The coagulation of small particles explains the particle diameter growth versus time while the total number of particles decreases.

#### Particle Mass Concentrations

A typical temporal evolution of the normalized mass concentrations (d*M*/dlogDp) is displayed in Figure 5b for the same initial concentrations as Figure 5a. In this example, the mass increase of the particles started 2 min after initiation of the T2H + Cl reaction. The mass concentrations of particles (*M*_SOA_) reached a plateau after roughly 30 min of reaction and then started to decrease, probably due to losses in the system.

#### SOA Yields

**Figure 6:**
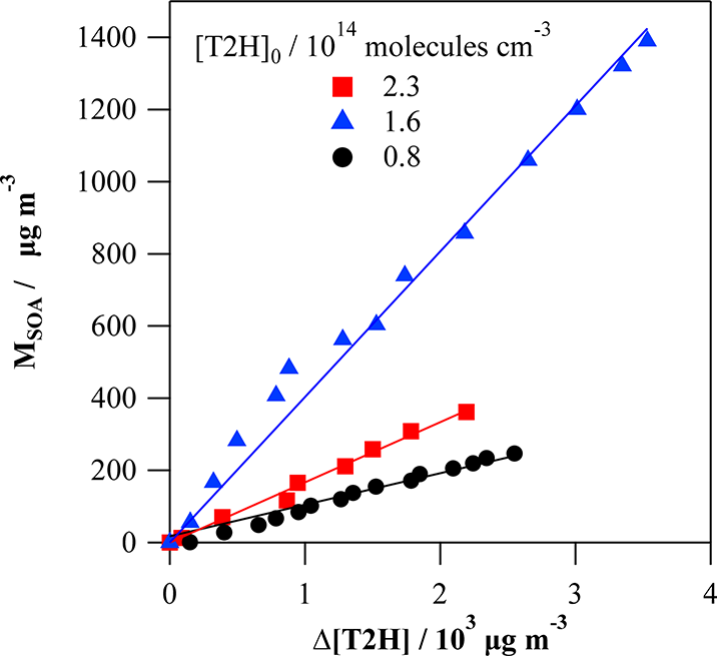
Examples of the produced SOA mass concentration *M*_SOA_ from the T2H + Cl reaction as a function of the consumed T2H (all initial concentrations of T2H and Cl_2_ are indicated in Table 3).

For every experimental initial condition, it is possible to determine the SOA yield *Y*_SOA_ from the slope of the *M*_SOA_ linear increase as a function of the T2H consumption as illustrated in Figure 6. Such plots were obtained for many different experimental conditions, and the SOA yields are given in Table 3 for each situation as well as the initial concentrations of T2H and Cl_2_ ([T2H]_0_ and [Cl_2_]_0_), the reacted T2H (Δ[T2H]), and *M*_SOA,max_.

**Table 3:** Experimental Conditions and Results Obtained for the SOA Study

[T2H]_0_ (10^–14^ molecules cm^–3^)	[Cl_2_]_0_ (10^–14^ molecules cm^–3^)	Δ[T2H] (10^–13^ molecules cm^–3^)	*M*_SOA,max_ (10^–2^ μg m^–3^)	% *Y*_SOA_ ± 2σ
1.4	1.3	1.1	1.8	5.1 ± 0.8
2.1	2.6	2.3	3.9	9.8 ± 1.1
0.8	2.1	1.7	2.5	10.5 ± 0.4
1.9	3.1	1.6	5.9	13.0 ± 2.4
2.2	4.3	2.0	7.1	15.3 ± 1.0
2.3	3.8	1.3	3.6	16.9 ± 1.4
2.2	5	1.7	7.0	20.7 ± 5.5
2.7	6.5	1.0	4.9	27.7 ± 4.8
2.5	6	2.5	15.3	29.9 ± 4.2
2.1	4.9	0.71	6.6	31.0 ± 4.6
1.2	5.7	1.7	15.7	31.1 ± 5.8
2.0	7.2	1.4	12.9	33.1 ± 2.8
1.1	4.0	0.7	5.2	33.1 ± 7.5
1.6	6.8	2.1	14.9	37.5 ± 4.4

*Y*_SOA_ for the T2H + Cl reaction has been found to range between 5 and 38%. These values are found to be higher than the results obtained for the Cl reaction of *trans*-2-pentenal, which were in the 1–7% range,^25^ and *trans*-2-methyl-2-butenal, which were between 0.3 and 1.7%.^41^ SOA yields in the range of 7 to 36%^53^ were obtained for the reaction of Cl with isoprene being these SOA yields more similar to the values determined in the present work for the reaction of Cl with T2H.

**Figure 7:**
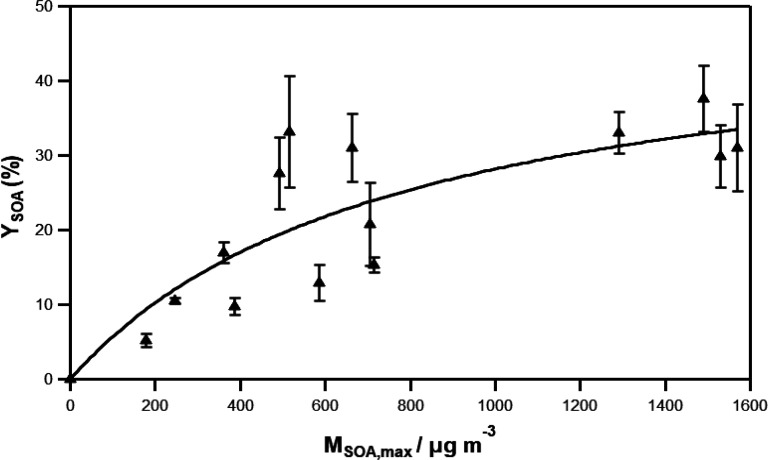
Plot of *Y*_SOA_ as a function of *M*_SOA,max_ for the reaction between T2H and Cl according to the one-product model developed by Odum et al.^54^ (eq 4).

Figure 7 shows the plot of *Y*_SOA_ versus *M*_SOA,max_, and the fit of the data using the one-product model from Odum et al.^54^ (eq 4).
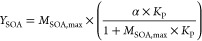
4where α is the mass-based gas-phase stoichiometric coefficient of a model product, and *K*_p_ represents its gas-particle partitioning equilibrium coefficient. A least-squares regression on the data gives the following parameters: α = (50.0 ± 13.5)% and *K*_p_ = (1.3 ± 0.7) × 10^–3^ m^3^ μg^–1^. Uncertainties represent two standard deviations (2σ). α indicates that *Y*_SOA_ tends toward 50% at very high *M*_SOA_ values, which is higher than what was found for a similar reaction, T2P + Cl,^25^ where α was 10%. On the other hand, *K*_p_ could indicate that the semivolatile species that produce the SOA formation are mostly in the vapor rather than the condensed phase, which is similar to what was observed for T2P + Cl (*K*_p_ = (6.0 ± 2.7) × 10^–4^ m^3^ μg^–1^, ref (25)). However, it must be noted that many products result from the oxidation of a VOC, so the parameters obtained from the fitting to eq 4 allow for easy comparison of yield curves, but as reported by Cai et al.,^55^ the fitted parameters provide no specific information about the real oxidation products, and ascribing meaning to these is not recommended.

## Atmospheric Implications and Conclusions

As discussed by Jiménez et al.,^21^ UV photodissociation of T2H in the solar actinic region is not a significant degradation process. Therefore, here we only consider the atmospheric lifetime (τ) of T2H due to the oxidation reactions with OH, Cl, O_3_, and NO_3_, which was estimated using the following equation:
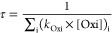
5where *k*_Oxi_ is the rate constant for the reaction of the unsaturated aldehyde with the oxidant (i) and [Oxi] is the averaged atmospheric concentration of the oxidant for two situations: a global atmosphere and a coastal area. Globally, the 24 h average concentrations considered to estimate τ were: [OH] = 1 × 10^6^ radicals cm^–3^,^56^ [Cl] = 1 × 10^3^ atoms cm^–3^,^57^ [O_3_] = 7 × 10^11^ molecules cm^–3^,^58^ and [NO_3_] = 2.5 × 10^8^ radicals cm^–3^.^59^ The individual atmospheric lifetimes calculated for every oxidant are summarized in Table 4. Globally, the depletion of T2H due to the Cl, O_3_, and NO_3_ reactions is negligible, since its individual lifetime is about 37, 10, and 3 days, respectively. As indicated in this table, the oxidation of T2H by OH reaction dominates the fate of T2H in the atmosphere with a lifetime τ_OH_ of 7 h. The overall lifetime calculated according to eq 5 is 6 h.

**Table 4:** Estimated Atmospheric Lifetimes of T2H toward Atmospheric Oxidants (Cl, O_3_, NO_3_, and OH)

*k*_Cl_ (10^–10^ molecule^–1^ cm^3^ s^–1^)	τ_Cl-high_ (h)	τ_Cl-low_ (days)	*k*_O3_ (10^–18^ molecule^–1^ cm^3^ s^–1^)	τ_O3_ (days)	k_NO3_ (10^–14^ molecule^–1^ cm^3^ s^–1^)	τ_NO3_ (days)	k_OH_ (10^–11^ molecule^–1^ cm^3^ s^–1^)	τ_OH_ (h)
3.17 ± 0.72^a^	9	37	**1.66**^b^	10	**1.71**^c^	3^c^	**3.88**^d^	7^d^

a This work.

b Average of Atkinson et al.,^23^ Grosjean et al.,^28^ Kalalian et al.,^29^ and Grira et al.^25^ (Table S3).

c Average of Grosjean et al.,^30^ Cabañas et al.,^31^ Zhao et al.,^32^ Kerdouci et al.,^33^ and Rayez et al.^34^ (Table S3).

d Average of Grosjean et al.,^30^ Atkinson et al.,^23^ Albaladejo et al.,^35^ Davis et al.,^22^ Jiménez et al.^21^ and Gao et al.^20^ (Table S3).

For the estimation of τ in the second scenario, the peak concentration measured at dawn in the marine boundary layer and polluted urban regions was used, [Cl] = 1 × 10^5^ atoms cm^–3^.^60^ In this specific situation, τ_Cl-high_ is 9 h. Therefore, the T2H + Cl reaction becomes competitive with the OH reaction, since at dawn, where the solar flux is very low, the OH concentration is on the order of magnitude of Cl atoms, 10^5^ radicals cm^–3^.^58^ The overall lifetime is 7 h in a chlorine enhanced concentration zone, with contributions of 10 and 77% of OH and Cl, respectively. Removal of T2H by NO_3_ and O_3_ are 10 and 3%, respectively, under this circumstance.

Regarding the impact of the T2H + Cl reaction on air quality, it may contribute to the local formation of secondary pollutants leading to the emission of free radicals (for example HO_2_ was formed from the α, β-addition, and the H-abstraction pathways) in the atmosphere. In the absence of NOx, as reported for the first time in the present work, the T2H + Cl reaction may significantly contribute to the harmful SOA formed (yields of up to 38%). This study was carried under NOx-free conditions; however, further studies are required to evaluate the contribution of NOx to the atmospheric degradation of unsaturated aldehydes.

## Supporting Information Available

Equations used in this work, figures concerning the kinetic and product studies, and tables summarizing rate constants (PDF)

## References

[ref1] Hatanaka A. The Fresh Green Odor Emitted by Plants. Food Rev. Int. 1996, 12 (3), 303–350. 10.1080/87559129609541083.

[ref2] Bourel G.; Nicaud J.-M.; Nthangeni B.; Santiago-Gomez P.; Belin J.-M.; Husson F. Fatty Acid Hydroperoxide Lyase of Green Bell Pepper: Cloning in Yarrowia Lipolytica and Biogenesis of Volatile Aldehydes. Enzyme Microb. Technol. 2004, 35 (4), 293–299. 10.1016/j.enzmictec.2003.12.014.

[ref3] Takeoka G. Flavor Chemistry of Vegetables. Flavor chemistry 1999, 287–304. 10.1007/978-1-4615-4693-1_25.

[ref4] Gray D. A.; Prestage S.; Linforth R. S.; Taylor A. J. Fresh Tomato Specific Fluctuations in the Composition of Lipoxygenase-Generated C_6_ Aldehydes. Food Chem. 1999, 64 (2), 149–155. 10.1016/S0308-8146(98)00163-0.

[ref5] Flavor and Aroma of Fresh-Cut Fruits and Vegetables. Fresh-Cut Fruits and Vegetables: Science, Technology, and Market; Lamikanra O., Ed.; CRC Press, 2002; pp 391–425.

[ref6] Poll L.; Nielsen G. S.; Varming C.; Petersen M. A. Aroma Changes from Raw to Processed Products in Fruits and Vegetables. Flavour Science - Recent Advances and Trends 2006, 43, 239–244. 10.1016/S0167-4501(06)80057-9.

[ref7] Bate N. J.; Rothstein S. J. C6-Volatiles Derived from the Lipoxygenase Pathway Induce a Subset of Defense-Related Genes. Plant J. 1998, 16 (5), 561–569. 10.1046/j.1365-313x.1998.00324.x. 10036774

[ref8] Gosset V.; Harmel N.; Göbel C.; Francis F.; Haubruge E.; Wathelet J.-P.; Du Jardin P.; Feussner I.; Fauconnier M.-L. Attacks by a Piercing-Sucking Insect (Myzus Persicae Sultzer) or a Chewing Insect (Leptinotarsa Decemlineata Say) on Potato Plants (Solanum Tuberosum L.) Induce Differential Changes in Volatile Compound Release and Oxylipin Synthesis. J. Exp. Bot. 2009, 60 (4), 1231–1240. 10.1093/jxb/erp015. 19221142 PMC2657539

[ref9] Fullana A.; Carbonell-Barrachina A. A.; Sidhu S. Comparison of Volatile Aldehydes Present in the Cooking Fumes of Extra Virgin Olive, Olive, and Canola Oils. J. Agric. Food Chem. 2004, 52 (16), 5207–5214. 10.1021/jf035241f. 15291498

[ref10] Katragadda H. R.; Fullana A.; Sidhu S.; Carbonell-Barrachina Á. A. Emissions of Volatile Aldehydes from Heated Cooking Oils. Food Chem. 2010, 120 (1), 59–65. 10.1016/j.foodchem.2009.09.070.

[ref11] Zhang Q.; Saleh A. S.; Chen J.; Shen Q. Chemical Alterations Taken Place during Deep-Fat Frying Based on Certain Reaction Products: A Review. Chem. Phys. Lipids 2012, 165 (6), 662–681. 10.1016/j.chemphyslip.2012.07.002. 22800882

[ref12] Esposto S.; Taticchi A.; Di Maio I.; Urbani S.; Veneziani G.; Selvaggini R.; Sordini B.; Servili M. Effect of an Olive Phenolic Extract on the Quality of Vegetable Oils during Frying. Food Chem. 2015, 176, 184–192. 10.1016/j.foodchem.2014.12.036. 25624222

[ref13] Blum M. S.; Crain R. D.; Chidester J. B. Trans-2-Hexenal in the Scent Gland of the Hemipteran Acanthocephala Femorata. Nature 1961, 189 (4760), 245–246. 10.1038/189245a0.

[ref14] Arey J.; Winer A. M.; Atkinson R.; Aschmann S. M.; Long W. D.; Lynn Morrison C. The Emission of (Z)-3-Hexen-1-Ol, (Z)-3-Hexenylacetate and Other Oxygenated Hydrocarbons from Agricultural Plant Species. Atmospheric Environ. Part Gen. Top. 1991, 25 (5–6), 1063–1075. 10.1016/0960-1686(91)90148-Z.

[ref15] König G.; Brunda M.; Puxbaum H.; Hewitt C. N.; Duckham S. C.; Rudolph J. Relative Contribution of Oxygenated Hydrocarbons to the Total Biogenic VOC Emissions of Selected Mid-European Agricultural and Natural Plant Species. Atmos. Environ. 1995, 29 (8), 861–874. 10.1016/1352-2310(95)00026-U.

[ref16] Kirstine W.; Galbally I.; Ye Y.; Hooper M. Emissions of Volatile Organic Compounds (Primarily Oxygenated Species) from Pasture. J. Geophys. Res. Atmospheres 1998, 103 (D9), 10605–10619. 10.1029/97JD03753.

[ref17] Fall R.; Karl T.; Hansel A.; Jordan A.; Lindinger W. Volatile Organic Compounds Emitted after Leaf Wounding: On-Line Analysis by Proton-Transfer-Reaction Mass Spectrometry. J. Geophys. Res. Atmospheres 1999, 104 (D13), 15963–15974. 10.1029/1999JD900144.

[ref18] Hatanaka A.; Harada T. Formation of Cis-3-Hexenal, Trans-2-Hexenal and Cis-3-Hexenol in Macerated Thea Sinensis Leaves. Phytochemistry 1973, 12 (10), 2341–2346. 10.1016/0031-9422(73)80435-2.

[ref19] Farag M. A.; Pare P. W. C6-Green Leaf Volatiles Trigger Local and Systemic VOC Emissions in Tomato. Phytochemistry 2002, 61 (5), 545–554. 10.1016/S0031-9422(02)00240-6. 12409021

[ref20] Gao T.; Andino J. M.; Rivera C. C.; Márquez M. F. Rate Constants of the Gas-Phase Reactions of OH Radicals with Trans-2-Hexenal, Trans-2-Octenal, and Trans-2-Nonenal. Int. J. Chem. Kinet. 2009, 41 (7), 483–489. 10.1002/kin.20424.

[ref21] Jiménez E.; Lanza B.; Martínez E.; Albaladejo J. Daytime Tropospheric Loss of hexanal and trans-2-hexenal: OH Kinetics and UV Photolysis. Atmospheric Chem. Phys. 2007, 7 (6), 1565–1574. 10.5194/acp-7-1565-2007.

[ref22] Davis M. E.; Gilles M. K.; Ravishankara A. R.; Burkholder J. B. Rate Coefficients for the Reaction of OH with (E)-2-Pentenal,(E)-2-Hexenal, and (E)-2-Heptenal. Phys. Chem. Chem. Phys. 2007, 9 (18), 2240–2248. 10.1039/b700235a. 17487321

[ref23] Atkinson; Arey J.; Aschmann S. M.; Corchnoy S. B.; Shu Y. Rate Constants for the Gas-Phase Reactions of Cis-3-Hexen-1-Ol, Cis-3-Hexenylacetate, Trans-2-Hexenal, and Linalool with OH and NO_3_ Radicals and O_3_ at 296 ± 2 K, and OH Radical Formation Yields from the O_3_ Reactions. Int. J. Chem. Kinet. 1995, 27 (10), 941–955. 10.1002/kin.550271002.

[ref24] Kalalian C.; Samir B.; Roth E.; Chakir A. UV Absorption Spectra of Trans-2-Pentenal, Trans-2-Hexenal and 2-Methyl-2-Pentenal. Chem. Phys. Lett. 2019, 718, 22–26. 10.1016/j.cplett.2019.01.028.

[ref25] Grira A.; Antiñolo M.; Canosa A.; Tomas A.; Jiménez E.; El Dib G. An Experimental Study of the Gas-Phase Reaction between Cl Atoms and Trans-2-Pentenal: Kinetics, Products and SOA Formation. Chemosphere 2021, 276, 130193 10.1016/j.chemosphere.2021.130193. 34088089

[ref26] Singh H. B.; Kasting J. F. Chlorine-Hydrocarbon Photochemistry in the Marine Troposphere and Lower Stratosphere. J. Atmospheric Chem. 1988, 7 (3), 261–285. 10.1007/BF00130933.

[ref27] Atkinson R.; Arey J. Atmospheric Degradation of Volatile Organic Compounds. Chem. Rev. 2003, 103 (12), 4605–4638. 10.1021/cr0206420. 14664626

[ref28] Grosjean E.; Grosjean D.; Seinfeld J. H. Gas-Phase Reaction of Ozone with Trans-2-Hexenal, Trans-2-Hexenyl Acetate, Ethylvinyl Ketone, and 6-Methyl-5-Hepten-2-One. Int. J. Chem. Kinet. 1996, 28 (5), 373–382. 10.1002/(SICI)1097-4601(1996)28:5<373::AID-KIN6>3.0.CO;2-S.

[ref29] Kalalian C.; Roth E.; Chakir A. Rate Coefficients for the Gas-Phase Reaction of Ozone with C_5_ and C_6_ Unsaturated Aldehydes. Int. J. Chem. Kinet. 2018, 50 (1), 47–56. 10.1002/kin.21139.

[ref30] Grosjean D.; Williams E. L. Environmental Persistence of Organic Compounds Estimated from Structure-Reactivity and Linear Free-Energy Relationships. Unsaturated Aliphatics. Atmospheric Environ. Part Gen. Top. 1992, 26 (8), 1395–1405. 10.1016/0960-1686(92)90124-4.

[ref31] Cabañas B.; Salgado S.; Martín P.; Baeza M. T.; Martínez E. Night-Time Atmospheric Loss Process for Unsaturated Aldehydes: Reaction with NO_3_ Radicals. J. Phys. Chem. A 2001, 105 (18), 4440–4445. 10.1021/jp0029459.

[ref32] Zhao Z.; Husainy S.; Smith G. D. Kinetics Studies of the Gas-Phase Reactions of NO_3_ Radicals with Series of 1-Alkenes, Dienes, Cycloalkenes, Alkenols, and Alkenals. J. Phys. Chem. A 2011, 115 (44), 12161–12172. 10.1021/jp206899w. 21995489

[ref33] Kerdouci J.; Picquet-Varrault B.; Durand-Jolibois R.; Gaimoz C.; Doussin J.-F. An Experimental Study of the Gas-Phase Reactions of NO_3_ Radicals with a Series of Unsaturated Aldehydes: Trans-2-Hexenal, Trans-2-Heptenal, and Trans-2-Octenal. J. Phys. Chem. A 2012, 116 (41), 10135–10142. 10.1021/jp3071234. 23004348

[ref34] Rayez M.-T.; Rayez J.-C.; Kerdouci J.; Picquet-Varrault B. Theoretical Study of the Gas-Phase Reactions of NO_3_ Radical with a Series of Trans-2-Unsaturated Aldehydes: From Acrolein to Trans-2-Octenal. J. Phys. Chem. A 2014, 118 (28), 5149–5155. 10.1021/jp503619d. 24987934

[ref35] Albaladejo J.; Ballesteros B.; Jiménez E.; Martín P.; Martínez E. A PLP–LIF Kinetic Study of the Atmospheric Reactivity of a Series of C_4_–C_7_ Saturated and Unsaturated Aliphatic Aldehydes with OH. Atmos. Environ. 2002, 36 (20), 3231–3239. 10.1016/S1352-2310(02)00323-0.

[ref36] Rodríguez D.; Rodríguez A.; Notario A.; Aranda A.; Díaz-de-Mera Y.; Martínez E. Kinetic Study of the Gas-Phase Reaction of Atomic Chlorine with a Series of Aldehydes. Atmospheric Chem. Phys. 2005, 5 (12), 3433–3440. 10.5194/acp-5-3433-2005.

[ref37] Teruel M. A.; Achad M.; Blanco M. B. Kinetic Study of the Reactions of Cl Atoms with α,β-Unsaturated Carbonyl Compounds at Atmospheric Pressure and Structure Activity Relations (SARs). Chem. Phys. Lett. 2009, 479 (1), 25–29. 10.1016/j.cplett.2009.07.103.

[ref38] Shashikala K.; Janardanan D. Degradation Mechanism of Trans-2-Hexenal in the Atmosphere. Chem. Phys. Lett. 2020, 759, 138039 10.1016/j.cplett.2020.138039.

[ref39] Turpin E.; Tomas A.; Fittschen C.; Devolder P.; Galloo J.-C. Acetone-H_6_ or -D_6_ + OH Reaction Products: Evidence for Heterogeneous Formation of Acetic Acid in a Simulation Chamber. Environ. Sci. Technol. 2006, 40 (19), 5956–5961. 10.1021/es060183a. 17051785

[ref40] Ballesteros B.; Jiménez E.; Moreno A.; Soto A.; Antiñolo M.; Albaladejo J. Atmospheric Fate of Hydrofluoroolefins, C_x_F_2x+1_CH=CH_2_ (x= 1, 2, 3, 4 and 6): Kinetics with Cl Atoms and Products. Chemosphere 2017, 167, 330–343. 10.1016/j.chemosphere.2016.09.156. 27736711

[ref41] Antiñolo M.; Asensio M.; Albaladejo J.; Jiménez E. Gas-Phase Reaction of Trans-2-Methyl-2-Butenal with Cl: Kinetics, Gaseous Products, and SOA Formation. Atmosphere 2020, 11 (7), 715 10.3390/atmos11070715. 33154821 PMC7116312

[ref42] Ragains M. L.; Finlayson-Pitts B. J. Kinetics and Mechanism of the Reaction of Cl Atoms with 2-Methyl-1,3-Butadiene (Isoprene) at 298 K. J. Phys. Chem. A 1997, 101 (8), 1509–1517. 10.1021/jp962786m.

[ref43] Fantechi G.; Jensen N. R.; Saastad O.; Hjorth J.; Peeters J. Reactions of Cl Atoms with Selected VOCs: Kinetics, Products and Mechanisms. J. Atmospheric Chem. 1998, 31 (3), 247–267. 10.1023/A:1006033910014.

[ref44] Orlando J. J.; Tyndall G. S.; Apel E. C.; Riemer D. D.; Paulson S. E. Rate Coefficients and Mechanisms of the Reaction of Cl-Atoms with a Series of Unsaturated Hydrocarbons under Atmospheric Conditions. Int. J. Chem. Kinet. 2003, 35 (8), 334–353. 10.1002/kin.10135.

[ref45] Hallquist M.; Wenger J. C.; Baltensperger U.; Rudich Y.; Simpson D.; Claeys M.; Dommen J.; Donahue N. M.; George C.; Goldstein A. H.; et al. The Formation, Properties and Impact of Secondary Organic Aerosol: Current and Emerging Issues. Atmos Chem. Phys. 2009, 9 (14), 5155–5236. 10.5194/acp-9-5155-2009.

[ref46] Antiñolo M.; del Olmo R.; Bravo I.; Albaladejo J.; Jiménez E. Tropospheric Fate of Allyl Cyanide (CH_2_=CHCH_2_CN): Kinetics, Reaction Products and Secondary Organic Aerosol Formation. Atmos. Environ. 2019, 219, 117041 10.1016/j.atmosenv.2019.117041.

[ref47] Thévenet R.; Mellouki A.; Le Bras G. Kinetics of OH and Cl Reactions with a Series of Aldehydes. Int. J. Chem. Kinet. 2000, 32 (11), 676–685. 10.1002/1097-4601(2000)32:11<676::AID-KIN3>3.0.CO;2-V.

[ref48] Canosa-Mas C. E.; Cotter E. S.; Duffy J.; Thompson K. C.; Wayne R. P. The Reactions of Atomic Chlorine with Acrolein, Methacrolein and Methyl Vinyl Ketone. Phys. Chem. Chem. Phys. 2001, 3 (15), 3075–3084. 10.1039/b101434j.

[ref49] Ullerstam M.; Ljungström E.; Langer S. Reactions of Acrolein, Crotonaldehyde and Pivalaldehyde with Cl Atoms: Structure–Activity Relationship and Comparison with OH and NO_3_ Reactions. Phys. Chem. Chem. Phys. 2001, 3 (6), 986–992. 10.1039/b007244n.

[ref50] Wang W.; Ezell M. J.; Ezell A. A.; Soskin G.; Finlayson-Pitts B. J. Rate Constants for the Reactions of Chlorine Atoms with a Series of Unsaturated Aldehydes and Ketones at 298 K: Structure and Reactivity. Phys. Chem. Chem. Phys. 2002, 4 (10), 1824–1831. 10.1039/b111557j.

[ref51] Mellouki A.; Le Bras G.; Sidebottom H. Kinetics and Mechanisms of the Oxidation of Oxygenated Organic Compounds in the Gas Phase. Chem. Rev. 2003, 103 (12), 5077–5096. 10.1021/cr020526x. 14664644

[ref52] Orlando J. J.; Tyndall G. S. Mechanisms for the Reactions of OH with Two Unsaturated Aldehydes: Crotonaldehyde and Acrolein. J. Phys. Chem. A 2002, 106 (51), 12252–12259. 10.1021/jp021530f.

[ref53] Wang D. S.; Ruiz L. H. Secondary Organic Aerosol from Chlorine-Initiated Oxidation of Isoprene. Atmospheric Chem. Phys. 2017, 17 (22), 13491–13508. 10.5194/acp-17-13491-2017.

[ref54] Odum J. R.; Hoffmann T.; Bowman F.; Collins D.; Flagan R. C.; Seinfeld J. H. Gas/Particle Partitioning and Secondary Organic Aerosol Yields. Environ. Sci. Technol. 1996, 30 (8), 2580–2585. 10.1021/es950943+.

[ref55] Cai X.; Griffin R. J. Secondary Aerosol Formation from the Oxidation of Biogenic Hydrocarbons by Chlorine Atoms. J. Geophys. Res. Atmospheres 2006, 111 (D14), D14206 10.1029/2005JD006857.

[ref56] Krol M.; van Leeuwen P. J.; Lelieveld J. Global OH Trend Inferred from Methylchloroform Measurements. J. Geophys. Res. Atmospheres 1998, 103 (D9), 10697–10711. 10.1029/98JD00459.

[ref57] Singh H. B.; Thakur A. N.; Chen Y. E.; Kanakidou M. Tetrachloroethylene as an Indicator of Low Cl Atom Concentrations in the Troposphere. Geophys. Res. Lett. 1996, 23 (12), 1529–1532. 10.1029/96GL01368.

[ref58] Finlayson-Pitts B. J.; Pitts J. N. Jr. Chemistry of the Upper and Lower Atmosphere: Theory, Experiments, and Applications; Elsevier, 2000.

[ref59] Calvert J.; Mechanisms of Atmospheric Oxidation of the Oxygenates; OUP, 2011.

[ref60] Spicer C. W.; Chapman E. G.; Finlayson-Pitts B. J.; Plastridge R. A.; Hubbe J. M.; Fast J. D.; Berkowitz C. M. Unexpectedly High Concentrations of Molecular Chlorine in Coastal Air. Nature 1998, 394 (6691), 353–356. 10.1038/28584.

