# Supplementary material for: Kinetic and Products Study of the Atmospheric Degradation of trans-2-Hexenal with Cl Atoms
Source: J Phys Chem A. 2022 Sep 27;126(39):6973–83. doi: 10.1021/acs.jpca.2c05060 (PMC9549468; doi:10.1021/acs.jpca.2c05060)
Supplement: Supplementary file 1 — jp2c05060_si_001.pdf [file jp2c05060_si_001.pdf]

Supporting Information

Kinetic and Products Study of the Atmospheric Degradation of *Trans*-2-hexenal with Cl Atoms

Asma Grira[1,2,*], María Antiñolo[3,4,*,#], André Canosa[1], Alexandre Tomas[2], Gisèle El Dib,[1] Elena Jiménez[3,4]

[1]*CNRS, IPR (Institut de Physique de Rennes)–UMR 6251. Université de Rennes, F–35000 Rennes, France*

[2]*IMT Nord Europe, Institut Mines–Télécom, Univ. Lille, Center for Energy and Environment, F–59000 Lille, France*

[3]*Departamento de Química Física, Facultad de Ciencias y Tecnologías Químicas, Universidad de Castilla–La Mancha, Avda. Camilo José Cela 1B, 13071, Ciudad Real, Spain*

[4]*Instituto de Investigación en Combustión y Contaminación Atmosférica (ICCA), Universidad de Castilla–La Mancha, Camino de Moledores s/n, 13071, Ciudad Real, Spain*

#Currently at: Escuela de Ingeniería Industrial y Aeroespacial, Universidad de Castilla–La Mancha, Avenida Carlos III s/n. Real Fábrica de Armas. 45071, Toledo, Spain.

*Corresponding authors:

Asma Grira. Phone: + 33 3 27 71 26 29; email: asma.grira@imt-nord-europe.fr
María Antiñolo. Phone: +34 9 26 29 53 00; email: maria.antinolo@uclm.es

Equations

Determination of Cl_2 concentration

The concentration of Cl_2 was determined based on the measurement of the pressure of gaseous Cl_2 injected in a calibrated volume. This volume was transferred afterwards to the reaction chamber, with a different volume. Then, the concentration of Cl_2 was determined using the following equation based on the ideal gas law:

$$[Cl_2]\left(\frac{molecules}{cm^3}\right) = \frac{P_{Cl_2}\,(atm) \times N_A\left(\frac{molecules}{mol}\right) \times Purity\,Cl_2\% \times V_{Cl_2}\,(L)}{R\left(\frac{atm\,L}{mol\,K}\right) \times T\,(K) \times V_{reactor}(cm^3)}$$

where:

- P_{Cl_2}: injected calibrated volume of Cl_2
- V_{Cl_2}: injected calibrated volume of Cl_2
- Purity Cl_2: 10% for the D-ASC and >99.8% for the CR-ASC and 16-L reactors.
- N_A: Avogadro constant ($N_A = 6.022 \times 10^{23}$ molecules mol^{-1})
- R: ideal gas constant (R = 0.082 atm L / (mol K))
- T: temperature
- $V_{reactor}$: volume of the reaction chamber

Determination of the overall error (Δk_{Iso}) in average k_{Iso}

The overall error on the rate constant $\boldsymbol{k_{Iso}}$ was calculated using equation Eq.S2.

$$\Delta k_{Iso} = \sqrt{\frac{\sum_{i=1}^{n}[w_i \times (k_{Iso,i} - <k_{Iso}>)^2]}{\frac{(n-1) \times \sum_{i=1}^{n} w_i}{n}}} \quad \text{(Eq.S2)}$$

where n is the number of data, w_i is the weighting factor (Eq.S3), which accounts for the standard deviation of individual Cl-rate constant for isoprene $k_{Iso,i}$ (σ_i), and $<k_{Iso}>$ is the weighted average rate constant given by equation Eq.S4.

$$w_i = \frac{1}{\sigma_i^2} \qquad \text{(Eq.S3)}$$

$$< k_{Iso} > \; = \frac{\sum_{i=1}^{n}(w_i \times k_{Iso,i})}{\sum_{i=1}^{n} w_i} \qquad \text{(Eq.S4)}$$

Determination of the overall error (Δk_{T2H}) in the average k_{T2H}

The overall error on $\boldsymbol{k_{T2H}}$ was calculated using the following equation:

$$\Delta k_{T2H} = \sqrt{\Delta k_{T2H}\,(\text{stat})^2 + \Delta k_{T2H}\,(\text{syst})^2} \qquad \text{(Eq.S5)}$$

- Systematic errors Δk_{T2H} (syst) due mainly to the difficulties in handling and measuring the concentrations of reagents with good accuracy were estimated to be 10% in this work. This includes the uncertainties in the measurement of the temperature and pressure that account for less than 1%.
- Statistical errors Δk_{T2H} (stat) were calculated according to the following equation:

$$\frac{\Delta k_{T2H}\,(\text{stat})}{k_{T2H}} = \frac{\Delta\left(\frac{k_{T2H}}{k_{Iso}}\right)}{\left(\frac{k_{T2H}}{k_{Iso}}\right)} + \frac{\Delta k_{Iso}}{k_{Iso}} \qquad \text{(Eq.S6)}$$

- $\Delta(k_{T2H}/k_{Iso})$ comes from the uncertainty in the slope of plots of (Eq.1) obtained by the least-squares analysis.

Determination of the overall error (ΔY_{Prod}) in the product yields

The overall error on the product formation yields were calculated using the following equation:

$$\Delta Y_{Prod} = \sqrt{\Delta Y_{Prod}\,(stat)^2 + \Delta Y_{Prod}\,(syst)^2} \qquad \text{(Eq.S7)}$$

- Systematic errors (ΔY_{Prod} (syst)) due mainly to the difficulties in handling and measuring the concentrations of reagents with good accuracy were estimated to be 10% in this work.
- Statistical errors ΔY_{Prod} (stat) result from a least-squares analysis of the $[Product]_t$ vs. $\Delta[T2H]$ plots (Eq.2).

Figures

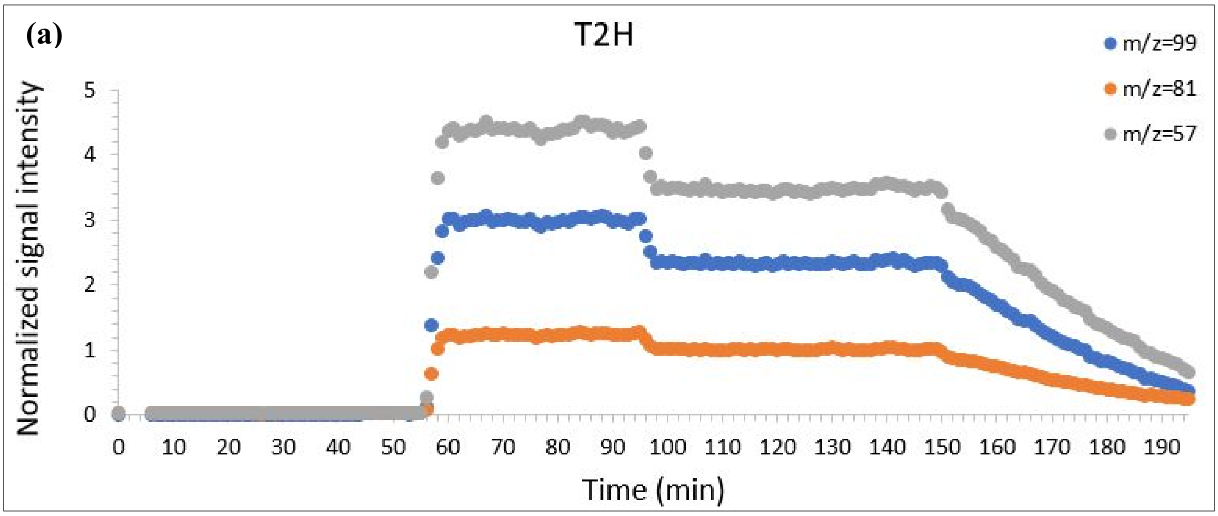

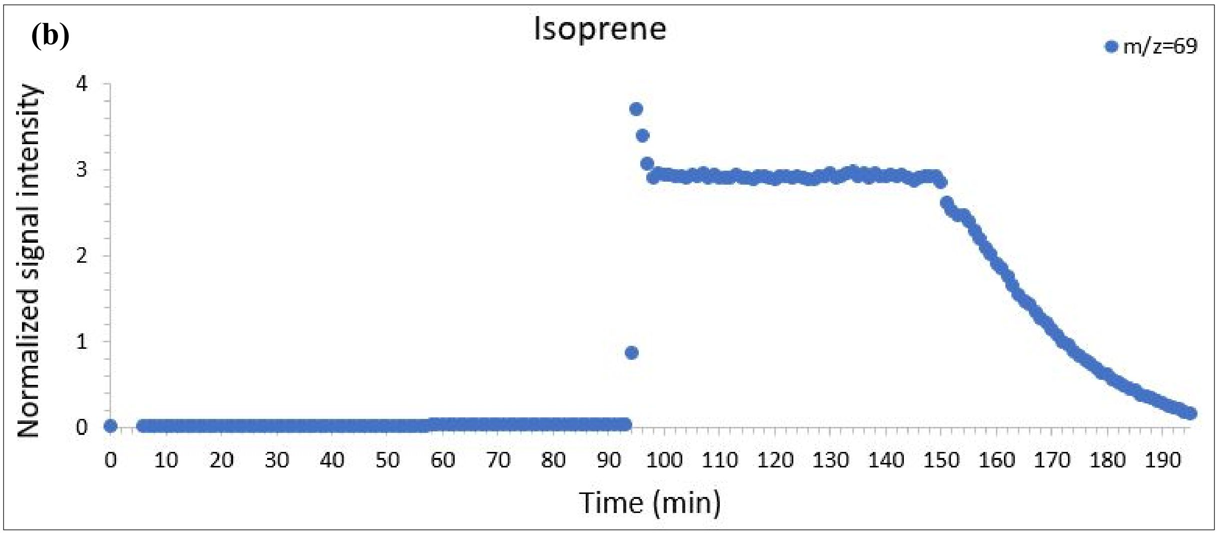

Figure S1. Plot of normalized PTR-MS signal profiles measured of (a) T2H and (b) isoprene in D-ASC (normalized to the signal of $H_3{}^{18}O^+$ ion, m/z = 21).

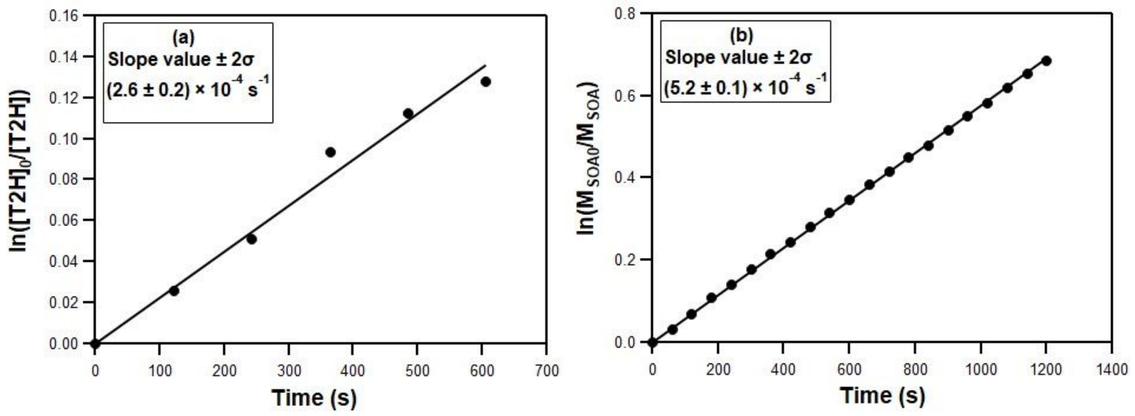

Figure S2. An example of wall losses of T2H (a) and SOA masses (b) $[T2H]_0 = 2.5\times10^{14}$ molecules cm^{-3} and $[Cl_2]_0 = 6.0\times10^{14}$ molecules cm^{-3}.

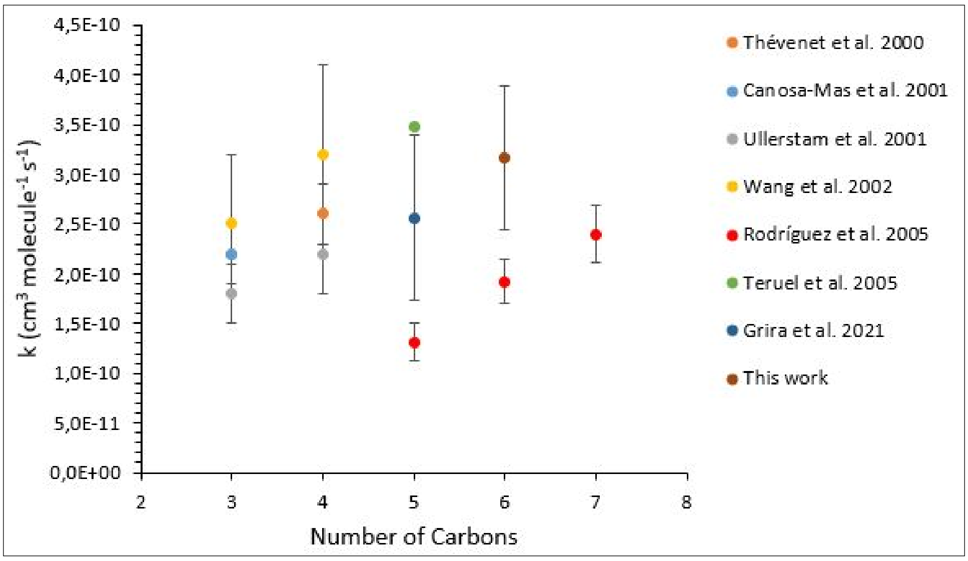

Figure S3. Plot of the rate constants of the reaction between C_3–C_7 alkenals and Cl as function of the carbon number.

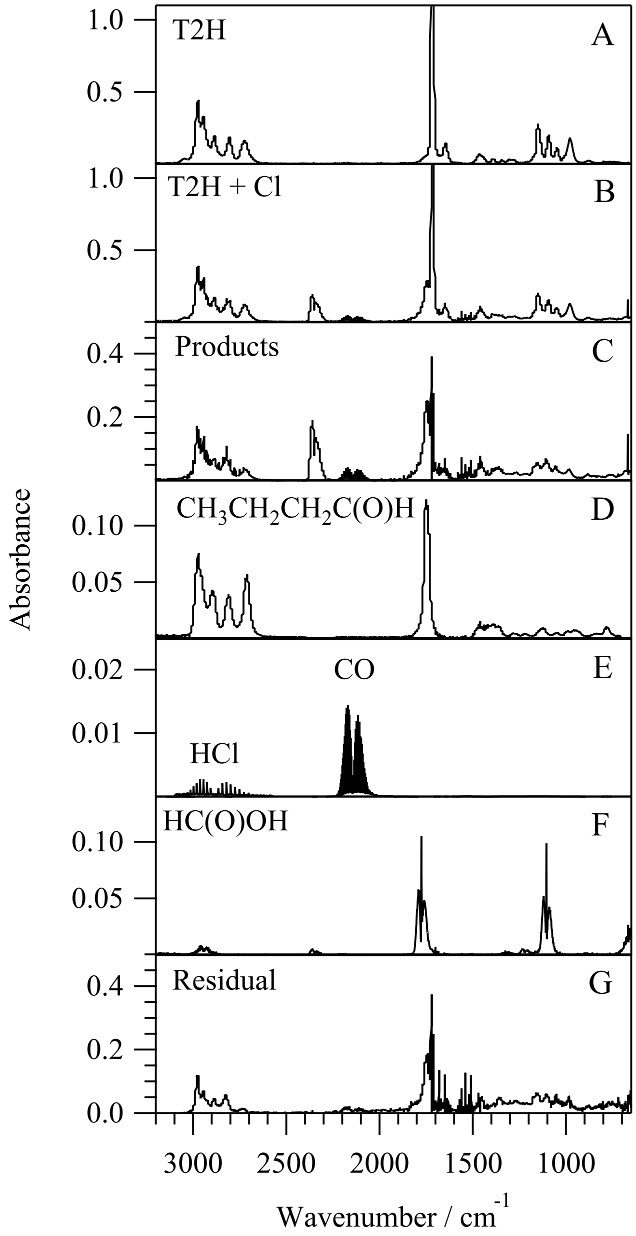

Figure S4. FTIR spectra. Panel A shows the T2H spectrum. Panel B shows the spectrum obtained after 10 min of Cl reaction of T2H. Panel C shows the spectrum in B with the features of T2H subtracted. Panels D-F show the reference spectra used in this work for the detected products. HCl, CO and HC(O)OH spectra were recorded in our lab. The IR spectrum of butanal was taken from the EUROCHAMP database[1]. Panel G shows the residual spectrum after the subtraction of the IR features corresponding to the detected products.

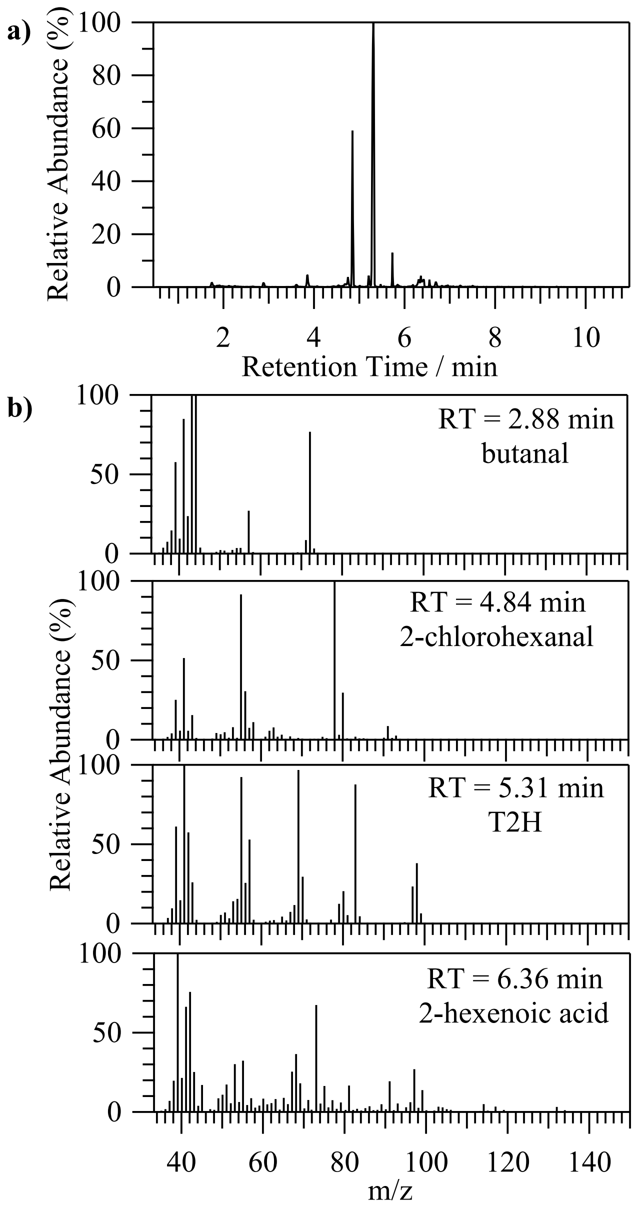

Figure S5. (a) Chromatogram obtained after 60 min of the Cl reaction of T2H. (b) Mass spectra of the most intense peaks of the chromatogram that were attributed to products. Other chromatogram peaks were due to impurities from the column or the fiber, or due to unidentified compounds.

Tables

Table S1. Summary of all rate constants (k_{T2H}) measured using T2H (m/z = 99, m/z = 81 and m/z = 57) and isoprene (m/z = 69).

Isoprene fragment	T2H fragments	k_{T2H}/k_{Iso}	k_{T2H} (10^{-10} cm^3 $molecule^{-1}$ s^{-1})
m/z = 69	m/z = 99	0.73	3.36
	m/z = 81	0.68	3.13
	m/z = 57	0.67	3.08

Table S2. Summary of literature rate constants of the reaction isoprene+Cl, the average value (k_{Iso}) and the overall error (Δk_{Iso}).

Compound	($k_{Iso}\pm\Delta k_{Iso}$) (10^{-10} cm^3 $molecule^{-1}$ s^{-1})	Reference
Isoprene	4.6±0.5	Ragains and Finlayson-Pitts [2]
	5.5±1.0	Fantechi et al.[3]
	4.3±0.6	Orlando et al.[4]
	**4.6±0.9**	**Used value (average ± 2σ)**

Table S3. Summary of the rate constants for the reactions of T2H with OH, O_3 and NO_3 at room temperature and atmospheric pressure. Uncertainties stated by the authors.

**T2H+OH**	
**k_{OH}±Δk (10^{-11} cm^3 molecule^{-1} s^{-1})**	**Reference**
3.10 *	Grosjean et al.[5] (estimated)
4.41±0.94 #	Atkinson et al.[6]
2.95±0.45 $	Albaladejo et al.[7]
4.47±0.50 $	Jiménez et al.[8]
4.40±0.5 $	Davis et al.[9]
3.93±0.17 $	Gao et al.[10]
**3.88** *	**Value used in Table 4**
**T2H+O_3**	
**k_{O_3}±Δk (10^{-18} cm^3 molecule^{-1} s^{-1})**	**Reference**
2.00±1.00 #	Atkinson et al.[6]
1.28±0.28 @	Grosjean et al.[11]
1.37±0.03 @	Kalalian et al.[12]
1.98±0.53 $	Grira et al.[13]
**1.66** *	**Value used in Table 4**
**T2H+NO_3**	
**k_{NO_3}±Δk (10^{-14} cm^3 molecule^{-1} s^{-1})**	**Reference**
1.70 *	Grosjean et al.[5] (estimated)
5.49±0.95 $	Cabañas et al.[14]
0.14±0.01 &	Zhao et al.[15]
0.47±0.01 $	Kerdouci et al.[16]

0.75 *	Rayez et al.[17](estimated)
**1.71 ***	**Value used in Table 4**

*No uncertainty provided. #Uncertainty is 2σ combined with the estimated overall uncertainties on k_{Ref}. $Uncertainty is 2σ. @Uncertainty is σ. &Uncertainty considers 95% confidence interval.

References

(1) Ródenas, M. IR Spectrum: BUTANAL || BUTYRALDEHYDE, **2017**.

(2) Ragains, M. L.; Finlayson-Pitts, B. J. Kinetics and Mechanism of the Reaction of Cl Atoms with 2-Methyl-1,3-Butadiene (Isoprene) at 298 K. J. Phys. Chem. A **1997**, 101 (8), 1509–1517

(3) Fantechi, G.; Jensen, N. R.; Saastad, O.; Hjorth, J.; Peeters, J. Reactions of Cl Atoms with Selected VOCs: Kinetics, Products and Mechanisms. J. Atmospheric Chem. **1998**, 31 (3), 247–267.

(4) Orlando, J. J.; Tyndall, G. S.; Apel, E. C.; Riemer, D. D.; Paulson, S. E. Rate Coefficients and Mechanisms of the Reaction of Cl-Atoms with a Series of Unsaturated Hydrocarbons under Atmospheric Conditions. Int. J. Chem. Kinet. **2003**, 35 (8), 334–353.

(5) Grosjean, D.; Williams, E. L. Environmental Persistence of Organic Compounds Estimated from Structure-Reactivity and Linear Free-Energy Relationships. Unsaturated Aliphatics. Atmospheric Environ. Part Gen. Top. **1992**, 26 (8), 1395–1405.

(6) Atkinson; Arey, J.; Aschmann, S. M.; Corchnoy, S. B.; Shu, Y. Rate Constants for the Gas-Phase Reactions of Cis-3-Hexen-1-Ol, Cis-3-Hexenylacetate, Trans-2-Hexenal, and Linalool with OH and NO_3 Radicals and O_3 at 296±2 K, and OH Radical Formation Yields from the O_3 Reactions. *Int. J. Chem. Kinet.* **1995**, *27* (10), 941–955.

(7) Albaladejo, J.; Ballesteros, B.; Jiménez, E.; Martín, P.; Martínez, E. A PLP–LIF Kinetic Study of the Atmospheric Reactivity of a Series of C_4–C_7 Saturated and Unsaturated Aliphatic Aldehydes with OH. *Atmos. Environ.* **2002**, *36* (20), 3231–3239.

(8) Jiménez, E.; Lanza, B.; Martínez, E.; Albaladejo, J. Daytime Tropospheric Loss of hexanal and trans-2-hexenal: OH Kinetics and UV Photolysis. *Atmospheric Chem. Phys.* **2007**, *7* (6), 1565–1574.

(9) Davis, M. E.; Gilles, M. K.; Ravishankara, A. R.; Burkholder, J. B. Rate Coefficients for the Reaction of OH with (E)-2-Pentenal,(E)-2-Hexenal, and (E)-2-Heptenal. *Phys. Chem. Chem. Phys.* **2007**, *9* (18), 2240–2248.

(10) Gao, T.; Andino, J. M.; Rivera, C. C.; Márquez, M. F. Rate Constants of the Gas-Phase Reactions of OH Radicals with Trans-2-Hexenal, Trans-2-Octenal, and Trans-2-Nonenal. *Int. J. Chem. Kinet.* **2009**, *41* (7), 483–489.

(11) Grosjean, E.; Grosjean, D.; Seinfeld, J. H. Gas-Phase Reaction of Ozone with Trans-2-Hexenal, Trans-2-Hexenyl Acetate, Ethylvinyl Ketone, and 6-Methyl-5-Hepten-2-One. *Int. J. Chem. Kinet.* **1996**, *28* (5), 373–382.

(12) Kalalian, C.; Roth, E.; Chakir, A. Rate Coefficients for the Gas-Phase Reaction of Ozone with C_5 and C_6 Unsaturated Aldehydes. *Int. J. Chem. Kinet.* **2017**, *50* (1), 47–56.

(13) Grira, A.; Antiñolo, M.; Canosa, A.; Tomas, A.; Jiménez, E.; El Dib, G. An Experimental Study of the Gas-Phase Reaction between Cl Atoms and Trans-2-Pentenal: Kinetics, Products and SOA Formation. *Chemosphere* **2021**, *276*, 130193.

(14) Cabanas, B.; Salgado, S.; Martin, P.; Baeza, M. T.; Martinez, E. Night-Time Atmospheric Loss Process for Unsaturated Aldehydes: Reaction with NO_3 Radicals. *J. Phys. Chem. A* **2001**, *105* (18), 4440–4445.

(15) Zhao, Z.; Husainy, S.; Smith, G. D. Kinetics Studies of the Gas-Phase Reactions of NO_3 Radicals with Series of 1-Alkenes, Dienes, Cycloalkenes, Alkenols, and Alkenals. *J. Phys. Chem. A* **2011**, *115* (44), 12161–12172.

(16) Kerdouci, J.; Picquet-Varrault, B.; Durand-Jolibois, R.; Gaimoz, C.; Doussin, J.-F. An Experimental Study of the Gas-Phase Reactions of NO_3 Radicals with a Series of Unsaturated Aldehydes: Trans-2-Hexenal, Trans-2-Heptenal, and Trans-2-Octenal. *J. Phys. Chem. A* **2012**, *116* (41), 10135–10142.

(17) Rayez, M.-T.; Rayez, J.-C.; Kerdouci, J.; Picquet-Varrault, B. Theoretical Study of the Gas-Phase Reactions of NO_3 Radical with a Series of Trans-2-Unsaturated Aldehydes: From Acrolein to Trans-2-Octenal. *J. Phys. Chem. A* **2014**, *118* (28), 5149–5155.
